# Inherited mitochondrial genetics as a predictor of immune checkpoint inhibition efficacy in melanoma

**DOI:** 10.1038/s41591-025-03699-3

**Published:** 2025-06-05

**Authors:** Kelsey R. Monson, Robert Ferguson, Joanna E. Handzlik, Leah Morales, Jiahan Xiong, Vylyny Chat, Sasha Dagayev, Alireza Khodadadi-Jamayran, Danny Simpson, Esther Kazlow, Anabelle Bunis, Chaitra Sreenivasaiah, Milad Ibrahim, Iryna Voloshyna, Wouter Ouwerkerk, Rosalie M. Luiten, Mariaelena Capone, Gabriele Madonna, Yuting Lu, Yongzhao Shao, Anna Pavlick, Michelle Krogsgaard, Janice Mehnert, Hao Tang, Sonia Dolfi, Daniel Tenney, John B. A. G. Haanen, Thomas F. Gajewski, F. Stephen Hodi, Keith T. Flaherty, Kasey Couts, William Robinson, Igor Puzanov, Marc S. Ernstoff, Osama Rahma, Michael Postow, Ryan J. Sullivan, Jason J. Luke, Paolo A. Ascierto, IO-GEM Consortium, Wouter Ouwerkerk, Rosalie M. Luiten, Mariaelena Capone, Gabriele Madonna, Anna Pavlick, John B. A. G. Haanen, Thomas F. Gajewski, F. Stephen Hodi, Keith T. Flaherty, Kasey Couts, William Robinson, Igor Puzanov, Marc S. Ernstoff, Osama Rahma, Michael Postow, Ryan J. Sullivan, Jason J. Luke, Paolo A. Ascierto, Iman Osman, Tomas Kirchhoff, Iman Osman, Tomas Kirchhoff

**Affiliations:** 1 Perlmutter Cancer Center, NYU Langone Health, New York, NY USA; 2 Departments of Population Health and Environmental Medicine, NYU Langone Health, New York, NY USA; 3 The Interdisciplinary Melanoma Cooperative Group, NYU Langone Health, New York, NY USA; 4 Tri-Institutional Training Program in Computational Biology and Medicine, Weill Cornell Medicine, New York, NY USA; 5 Applied Bioinformatics Laboratories, Office of Science and Research, New York University School of Medicine, New York, NY USA; 6 New York Genome Center, New York, NY USA; 7 Department of Medicine, NYU Langone Health, New York, NY USA; 8 Ronald O. Perelman Department of Dermatology, NYU Langone Health, New York, NY USA; 9 Department of Pathology, New York University Grossman School of Medicine, New York, NY USA; 10 Department of Dermatology and Netherlands Institute for Pigment Disorders, Amsterdam UMC, University of Amsterdam, Amsterdam Institute for Immunology & Infectious Diseases, Cancer Center, Amsterdam, the Netherlands; 11 Melanoma Cancer Immunotherapy and Development Therapeutics Unit, Istituto Nazionale Tumori IRCCS Fondazione G. Pascale, Naples, Italy; 12 Division of Hematology & Medical Oncology, the Cutaneous Oncology Program, Weill Cornell, New York, NY USA; 13 Bristol Myers Squibb Corp, Princeton, NJ USA; 14 Medical Oncology, Antoni van Leeuwenhoek Nederlands Kanker Instituut, Amsterdam, the Netherlands; 15 Department of Pathology, University of Chicago, Chicago, IL USA; 16 Section of Hematology/Oncology, Department of Medicine, University of Chicago, Chicago, IL USA; 17 Ben May Department for Cancer Research, University of Chicago, Chicago, IL USA; 18 Department of Medical Oncology, Center for Immuno-Oncology, Dana-Farber Cancer Institute, Boston, MA USA; 19 Center for Melanoma, Massachusetts General Hospital Cancer Center, Harvard Medical School, Boston, MA USA; 20 Department of Medicine, Division of Medical Oncology, University of Colorado, Aurora, CO USA; 21 Department of Medicine, Roswell Park Comprehensive Cancer Center, Buffalo, NY USA; 22 ImmunoOncology Branch (IOB), Developmental Therapeutics Program, Cancer Therapy and Diagnosis Division, National Cancer Institute (NCI), National Institutes of Health (NIH), Bethesda, MD USA; 23 Department of Medicine, Memorial Sloan Kettering Cancer Center, New York, NY USA; 24 Department of Medicine, Weill Cornell Medical College, New York, NY USA; 25 Department of Medicine, University of Pittsburgh, Pittsburgh, PA USA; 26 UPMC Hillman Cancer Center, Pittsburgh, PA USA

**Keywords:** Melanoma, Predictive markers, Immunotherapy, Genetic markers

## Abstract

Response to immune checkpoint inhibitors (ICIs) in metastatic melanoma (MM) varies among patients, and current baseline biomarkers predicting treatment outcomes are limited. As mitochondrial (MT) metabolism has emerged as an important regulator of host immune function, we explored the association of host MT genetics (MT haplogroups) with ICI efficacy in 1,225 ICI-treated patients with MM from the clinical trial CheckMate-067 and the International Germline Immuno-Oncology Melanoma Consortium. We discovered and validated significant associations of MT haplogroup T (HG-T) with resistance to anti-programmed cell death protein-1-based ICI (both single-agent and combination) and have shown that HG-T is independent from established tumor predictors. We also found that patients belonging to HG-T exhibit a unique nivolumab-resistant baseline peripheral CD8^+^ T cell repertoire compared to other MT haplogroups, providing, to our knowledge, the first link between MT inheritance, host immunity and ICI resistance. The study proposes a host blood-based biomarker with stand-alone clinical value predicting ICI efficacy and points to an ICI-resistance mechanism associated with MT metabolism, with clinical relevance in immuno-oncology.

## Abstract (web summary)

In this genomic analysis of peripheral blood samples of the phase 3 CheckMate-067 trial of ipilimumab (IPI) versus nivolumab (NIVO) versus ipilimumab and nivolumab (IPI-NIVO) in melanoma, the status of certain mitochondrial haplogroups in patients was associated therapeutic resistance to NIVO or IPI-NIVO, a finding validated in an independent cohort.

## Main

ICIs, either single-agent anti-programmed cell death protein-1 (PD-1) or in combination with anti-CTLA-4 (refs. ^1,2^) or other immune checkpoints^3^, are approved first-line therapies for MM. While these treatments produce durable responses, ~50% of ICI-treated patients still die of disease^4^. With the current portfolio of ICI treatments and the rapid development of novel ICI combinations to further improve patient outcomes, an important clinical need in immuno-oncology is the availability of reliable pretreatment biomarkers predicting ICI efficacy or informing patient selection for the most beneficial treatment options.

Existing tumor-based ICI outcome predictors, such as tumor mutational burden (TMB)^5,6^ or tumor programmed death ligand 1 (PD-L1) status^7^, alone or in combination, do not sufficiently explain the heterogeneity of the observed outcomes, let alone enable more personalized therapy selection. The complexities of tumor-based assessment further complicate their clinical applicability. In contrast, the host preexisting immune contexture has emerged as an important indicator of antitumor immune response to ICI. Nevertheless, only limited efforts have been expended to date to investigate the relevance of host immune predictors. For example, immune cell states^8,9^, gut microbiota^10,11^ or germline genetic variants^12,13^ have all been proposed as surrogates of ICI efficacy in melanoma; however, none of these has yet reached clinical translation for optimizing therapy selection.

Recent data suggest that host peripheral effector T cells rely on mitochondrially mediated metabolic signaling for activation and clonal expansion, in particular via oxidative phosphorylation (OXPHOS)^14^. It was proposed that OXPHOS produces a rapid increase in mitochondrially derived reactive oxygen species (ROS)^15^, which stimulate the proliferation of T cells that can be reinvigorated by disrupting the PD-1–PD-L1 axis upon anti-PD-1 blockade, thereby improving tumor killing^16^. However, one proposed mechanism of ICI resistance in treated patients is the abundance of OXPHOS-high terminally exhausted CD8^+^ T cells, which have lost the capacity for anti-PD-1 reinvigoration^14^, exhibiting disruption in MT function. ROS metabolism has been associated with inherited genetic differences in mitochondrial DNA (mtDNA), known as mitochondrial haplogroups (MT-HGs)^17^. Passed on through matrilineal inheritance, MT-HGs represent major branch points in the MT phylogenetic tree as humans dispersed globally from Africa over evolutionary time. In our study, given the growing evidence that MT pathways may impact T cell development and antitumor immunity^14,18^, we tested whether MT-HGs represent a novel host genetic biomarker of ICI outcome.

Using peripheral blood samples from CheckMate-067 (CM-067)^2^, a phase III MM clinical trial with extensive annotated clinical information, we assessed if response to anti-CTLA-4 (ipilimumab; IPI), anti-PD-1 (nivolumab; NIVO) and their combination (IPI-NIVO or COMBO) differs by MT-HG status. We found that resistance to single-agent NIVO and COMBO treatments is associated with specific MT-HGs, corresponding to ICI-resistant peripheral cell phenotypes in these patients. The associations of MT-HGs with ICI efficacy were validated in the standard-of-care (SOC) setting using patient samples collected by the International Germline Immuno-Oncology Melanoma Consortium (IO-GEM).

## Results

### MT-HGs are associated with efficacy in NIVO-treated patients

**Fig. 1:**
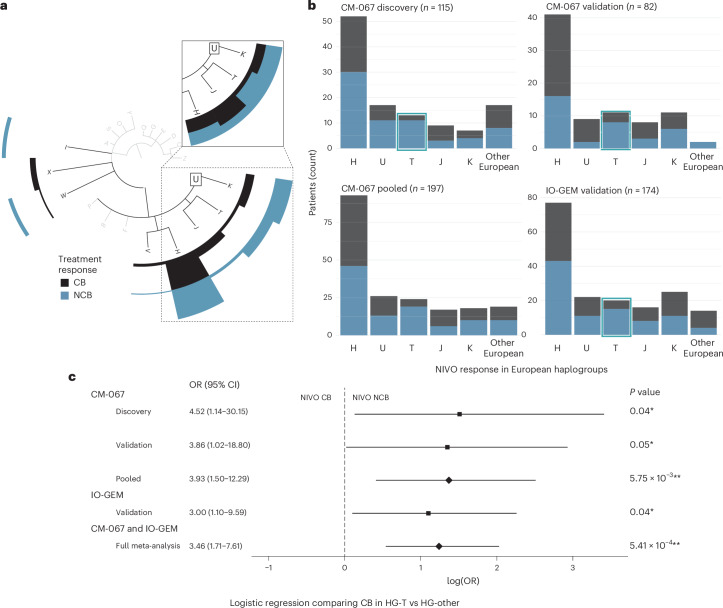
NIVO treatment efficacy by European haplogroup. Dark bars represent participants with NIVO CB (CR, PR or dSD). Light bars represent participants with NIVO NCB (pSD or PoD). **a**, Phylogenetic tree depicting MT-HG lineages and CM-067 NIVO discovery cohort treatment efficacy (*n* = 115 patients) with CB/NCB frequencies (counts) for the major European haplogroups (H, U/K, T, J, V, I, X, W). Inset shows CB/NCB proportions within each major European haplogroup (H, J, T, U/K). **b**, CM-067 and IO-GEM NIVO treatment efficacy frequencies by European haplogroup (H, U, T, J, K and Other, including W, R, V, X, I). Left to right (from top left), CM-067 NIVO discovery cohort (*n* = 115), CM-067 NIVO validation cohort (*n* = 82), CM-067 pooled (*n* = 197) and IO-GEM validation cohort (*n* = 174). **c**, Forest plot from logistic regression analysis showing the ORs, log odds, CIs and two-tailed *P* values when comparing NIVO treatment outcomes for HG-T versus other MT-HGs. Black boxes represent the point estimate for the main effect size (log odds) in discovery and validation cohorts and the lines extending from them represent the 95% CIs. Diamonds represent the log odds point estimate for pooled analyses estimating the overall magnitude of effect. Point estimates to the left of the dashed line indicate increased odds of NIVO CB, while point estimates to the right indicate increased odds of no NIVO CB. CIs that do not cross the dashed line indicate a statistically significant point estimate (*P* < 0.05). Source data

**Extended Data Fig. 1:**
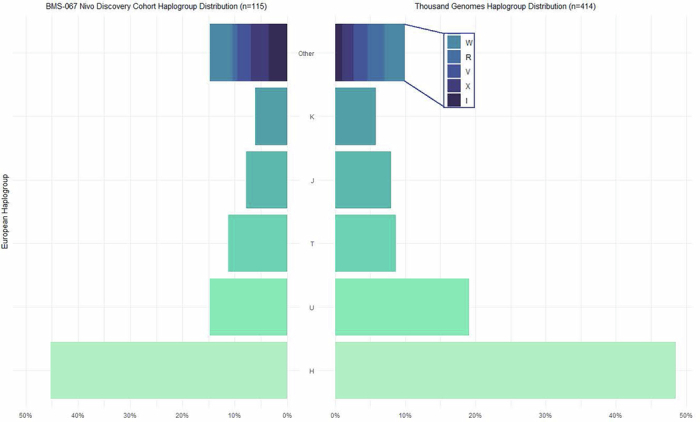
Percentage of subjects in each European haplogroup. Haplogroup distributions comparing subjects in BMS-067 Nivo Discovery Cohort (left; n=115) and subjects with European ancestry from the 1000 Genomes Project (right; n=414). Most frequent European haplogroups (H, U, T, J, and K) are plotted individually, with less common haplogroups (W, R, V, X, I) grouped together as “Other”.

In this study, we tested whether MT-HGs, as inherited genetic factors, are associated with ICI clinical efficacy in MM, and whether MT-HGs can be proposed as host predictive biomarkers of ICI treatment outcome. We first assessed association of MT-HG status with ICI clinical benefit (CB) or no clinical benefit (NCB) in 115 patients with MM enrolled in the CM-067 clinical trial who received NIVO. In this discovery cohort (CM-067-D), 48 NIVO-treated patients (42%) showed CB, experiencing either a complete or partial response or durable stable disease (dSD; Supplementary Table 1). The distribution of MT-HGs in the CM-067-D NIVO-treated cohort is as expected in the European population (Extended Data Fig. 1 and Supplementary Table 2). Similarly to the European population frequency (~50%), the most common MT-HG in the CM-067-D NIVO cohort was HG-H (46%), and 42% of patients with HG-H showed CB (Fig. 1a,b).

We saw significant differences in NIVO efficacy for patients with the HG-T in the CM-067-D NIVO cohort (~10% of both the European and CM-067-D NIVO cohort; Fig. 1a,b). Fifteen percent of patients belonging to HG-T showed NIVO CB, compared to 45% of patients with ‘other’ haplogroups (*P* value = 0.04), with significantly higher odds of NCB (odds ratio (OR) for NCB, T versus other MT-HGs = 4.52 (95% confidence interval (CI): 1.14–30.2); Fig. 1c). To validate these associations, we analyzed an additional *n* = 82 NIVO-treated patients from the CM-067 trial (CM-067-V; Supplementary Table 1). We again found a strong association between NIVO NCB and HG-T (*P* value = 0.048), with a 73% NCB rate in patients belonging to HG-T (OR for NCB, T versus other MT-HGs = 3.86 (95% CI: 1.02–18.8); Fig. 1b,c).

**Extended Data Fig. 2:**
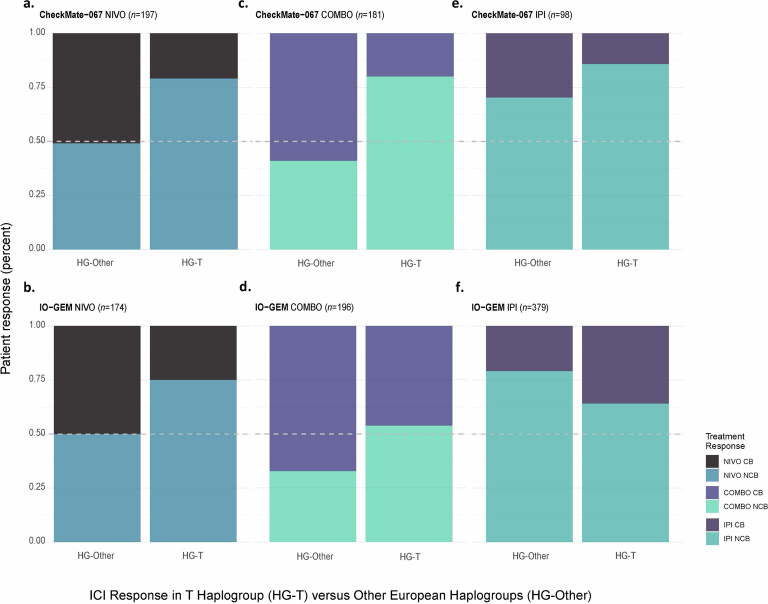
Response to ICI by haplogroup in CM-067 and IO-GEM cohorts. Proportion of CB and NCB ICI treatment responses in NIVO, COMBO (IPI-NIVO), and IPI cohorts comparing HG-T and other (H, U, J, K, W, R, V, X, and I) MT-HGs in: (**a**) CM-067 NIVO cohort (n=197), two-tailed Chi-square p-value = 5.75E-03; (**b**) IO-GEM NIVO cohort (n=174), two-tailed Chi-square p-value = 0.04; (**c**) CM-067 COMBO cohort (n=181), two-tailed Chi-square p-value = 3.55E-03; (**d**) IO-GEM COMBO cohort (n=196), two-tailed Chi-square p-value = 0.12; (**e**) CM-067 IPI cohort (n=98), two-tailed Chi-square p-value = 0.39; (**f**) IO-GEM IPI cohort (n=379), two-tailed Chi-square p-value = 0.03; Total CheckMate-067, n=476; Total IO-GEM, n=749.

To provide a robust estimate of the overall effect magnitude of the observed association in the setting of a highly controlled clinical trial, we combined all CM-067 NIVO-treated patients (*n* = 197) into a pooled analysis. Overall, 80% of NIVO-treated patients belonging to HG-T from CM-067 experienced NCB, compared to 51% CB in other MT-HGs (OR for NCB, T versus other MT-HGs = 3.93 (95% CI: 1.50–12.3), *P* value = 5.75 × 10^−3^; Fig. 1b,c and Extended Data Fig. 2a). Adjusting the logistic regression model for age and disease stage further refined the association of HG-T with NCB (adjusted OR for NCB, T versus other MT-HGs = 4.05 (95% CI: 1.53–12.8)).

To validate these findings in the SOC setting, we used samples obtained from the IO-GEM, a collaborative international consortium of immunotherapy-treated patients with melanoma (*n* = 113 NIVO samples). We also included data derived from previously published datasets (*n* = 61)^19,20^. Clinical characteristics and MT-HG distribution of the IO-GEM NIVO cohort (*n* = 174) are consistent with those seen in the CM-067 analytical cohort (Supplementary Tables 1 and 2). Overall efficacy in the IO-GEM NIVO cohort is similar to the CM-067 analysis, with 47% of participants experiencing CB (Fig. 1b).

The IO-GEM analyses confirmed the observed associations from CM-067, showing that HG-T is significantly associated with NIVO resistance (75% NCB rate, *P* value = 0.04; OR for NCB, T versus other MT-HGs = 3.00 (95% CI: 1.10–9.59)). This association is comparable with the effect size and the significance observed in the discovery analysis in CM-067 (Fig. 1b,c and Extended Data Fig. 2b). Adjusting for age and sex, the association remains comparably significant (OR = 2.78, 95% CI: 1.00–9.15).

The meta-analysis, performed solely to evaluate the overall magnitude of the effect by combining all NIVO-treated patients (CM-067 and IO-GEM, *n* = 371), demonstrated a strong overall association with NIVO resistance (OR for NCB, T versus other MT-HGs = 3.46 (95% CI: 1.71–7.61), *P* value = 5.41 × 10^−4^; Fig. 1c), and the association remained after adjusting for age and sex (adjusted OR for NCB, T versus other MT-HGs = 3.43 (95% CI: 1.69–7.57)). A sensitivity analysis assessing the objective response by comparing only complete response (CR) or partial response (PR) to patients with progression of disease (PoD), removing those with stable disease (SD), showed the same association trend with comparable statistical significance (OR for PoD, T versus other MT-HGs = 2.86, 95% CI: 1.39–6.38; *P* value = 4.86 × 10^−3^).

### MT-HGs predict clinical efficacy in other ICI regimens

**Fig. 2:**
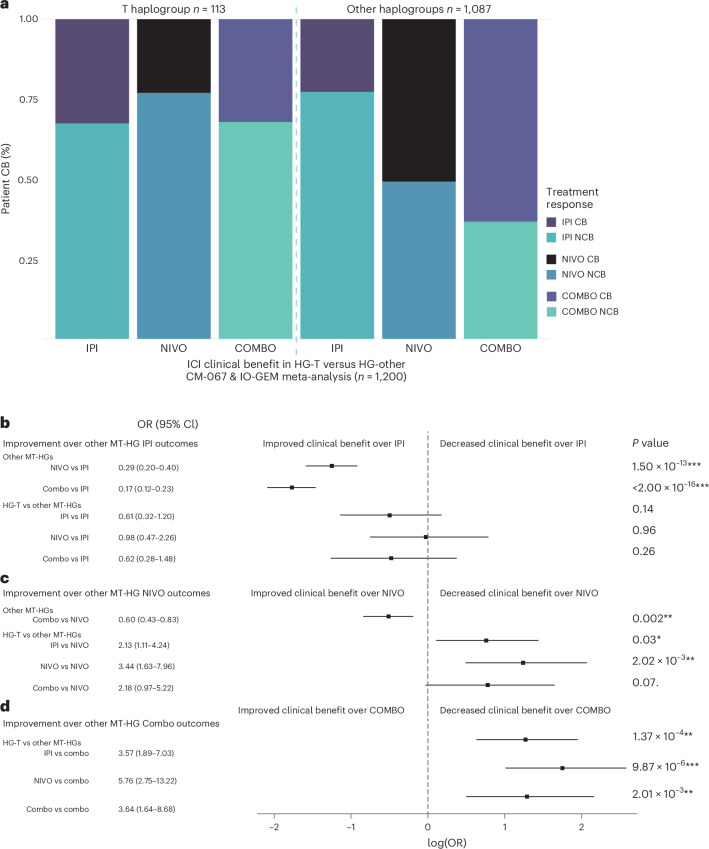
ICI treatment efficacy in IPI, NIVO or IPI-NIVO cohorts comparing HG-T and other (H, U, J, K, W, R, V, X and I) MT-HGs. **a**, Meta-analysis proportions of CB and NCB comparing HG-T (*n* = 113) and other MT-HGs (*n* = 1,087) for CM-067 and IO-GEM IPI cohort (CTLA-4); CM-067 and IO-GEM NIVO cohort (PD-1); CM-067 and IO-GEM COMBO cohort. **b**–**d**, Forest plots from logistic regression analysis illustrating the improvement in ICI outcomes across treatments for HG-T and other MT-HGs for the patient population in **a**, showing the ORs, log odds, CIs and two-tailed *P* values. When a treatment is shown to be a significant improvement over another, it is not presented again in subsequent comparisons. Comparison groups are as follows: other MT-HGs treated with IPI (*n* = 431), NIVO (*n* = 307) or COMBO (*n* = 349); HG-T treated with IPI (*n* = 46), NIVO (*n* = 39) or COMBO (*n* = 28). **b**, Logistic regression comparing ICI treatment outcomes to outcomes for other MT-HGs treated with IPI (*n* = 431); point estimates to the left of the dashed line indicate an improvement in outcomes over those of other MT-HGs treated with IPI. For example, for other MT-HGs, treatment outcomes in NIVO were a significant improvement over treatment outcomes in IPI (row 1), while there was no statistical difference in treatment outcomes for patients belonging to HG-T who were treated with NIVO compared to other MT-HGs treated with IPI (row 4). **c**, Logistic regression comparing ICI treatment outcomes to outcomes for other MT-HGs treated with NIVO (*n* = 307). **d**, Logistic regression comparing ICI treatment outcomes to outcomes for other MT-HGs treated with COMBO (*n* = 349). Black boxes represent the point estimate for the main effect size (log odds) and the lines extending from them represent the 95% CIs. Point estimates to the left of the dashed line indicate increased odds of ICI CB compared to the reference (patients belonging to other European haplogroups who were treated with IPI, NIVO or COMBO), while point estimates to the right indicate increased odds of no ICI CB compared to the reference. CIs that do not cross the dashed line indicate a statistically significant point estimate (*P* < 0.05). CB (CR, PR, dSD); NCB (pSD, PoD). Source data

To evaluate whether MT-HGs associate with outcomes of other frontline ICI therapies in MM, we tested MT-HG associations with the clinical efficacy of IPI-NIVO combination (anti-CTLA-4/anti-PD-1; COMBO), which is currently an approved SOC first-line ICI regimen for patients with MM. In CM-067 (*n* = 181), COMBO-treated patients belonging to HG-T showed significantly worse efficacy compared to all other MT-HGs (*P* value = 3.55 × 10^−3^), with an 80% NCB rate (OR for NCB, T versus other MT-HGs = 5.76 (95% CI: 1.75–26.0); Extended Data Fig. 2c), and this association remained comparably significant when adjusting for age and sex (OR = 5.73, 95% CI: 1.71–26.1). The same trend was shown in the IO-GEM COMBO cohort (*n* = 196), with patients belonging to HG-T showing the poorest outcomes after COMBO (54% NCB versus 33% NCB in other MT-HGs; Supplementary Table 1; OR for NCB, T versus other MT-HGs = 2.39 (95% CI: 0.76–7.73), *P* value = 0.12; Extended Data Fig. 2d), with comparable effect size when adjusting for age and sex (OR = 2.65, 95% CI: 0.79–9.19). To estimate the overall magnitude of the effect in the COMBO-treated patient population, we combined both CM-067 and IO-GEM COMBO-treated patients (*n* = 377), and found a strong association, with a 68% NCB rate in patients belonging to HG-T compared to a 34% NCB rate in the other MT-HGs (OR for NCB, T versus other MT-HGs = 3.64 (95% CI: 1.64–8.68), *P* value = 1.14 × 10^−3^; Fig. 2a). Adjusting the COMBO meta-analysis for age and sex demonstrated the same trend (adjusted OR = 3.64 (95% CI: 1.61–8.85)), as did a sensitivity analysis assessing objective response (OR for PoD, T versus other MT-HGs = 3.14, 95% CI: 1.33–7.80; *P* value = 7.28 × 10^−3^).

We also tested whether MT-HGs are associated with single-agent IPI (anti-CTLA-4) outcomes. There was no association between HG-T outcomes in the CM-067 IPI cohort (Extended Data Fig. 2e) compared to the IO-GEM IPI cohort (Extended Data Fig. 2f), and the meta-analysis combining both CM-067 and IO-GEM (*n* = 477) showed no statistically significant association of IPI efficacy with any of the MT-HGs (adjusted OR = 0.59 (95% CI: 0.31–1.18), *P* value = 0.13; Fig. 2a). We then evaluated improvements in outcomes for the different MT-HGs and ICI treatments to quantify the magnitude of the difference in CB for each treatment and haplogroup. For non-T MT-HGs, when compared to IPI, we found statistically significant improved outcomes in NIVO with further improvement in COMBO (OR = 0.29 and 0.17, respectively; Fig. 2b). For HG-T, however, none of the tested treatment regimens (IPI, NIVO or COMBO) showed statistically significant improvement when compared to non-T MT-HGs treated by IPI (Fig. 2b). For non-T MT-HGs, when compared to NIVO, COMBO-treated patients show significant improvement (OR = 0.60; Fig. 2c). The patients belonging to HG-T, however, when compared to non-T MT-HGs treated by NIVO, show comparably poorer outcomes for all three tested regimens (IPI, NIVO and COMBO; OR = 2.13, 3.44 and 2.18, respectively; Fig. 2c). The poor response outcomes observed in patients belonging to HG-T in all three regimens (IPI, NIVO and COMBO) were most significantly pronounced when compared to COMBO-treated patients belonging to non-T MT-HGs (OR = 3.57, 5.76 and 3.64, respectively; Fig. 2d).

### HG-T subgroups do not correlate with NIVO response

HG-T, defined by the presence of ten ‘parental’ single nucleotide polymorphisms (SNPs), has been well characterized as consisting of several subgroups^21^, further defined by additional variants. We explored whether resistance to NIVO-based ICI was associated with the two primary subtypes of HG-T, T1 and T2. While subgroup T2 was more prevalent (~70% of all HG-Ts in our patient population), there was no statistical difference in response rates between HG-T1 and HG-T2 in NIVO (69% NCB in T1, 83% NCB in T2; *P* value = 0.30) or COMBO (71% NCB in T1, 67% NCB in T2; *P* value = 0.82) or a pooled analysis (NIVO and COMBO; 70% NCB in T1, 76% NCB in T2; *P* value = 0.57).

### The predictive value of HG-T versus tumor-based ICI markers

**Fig. 3:**
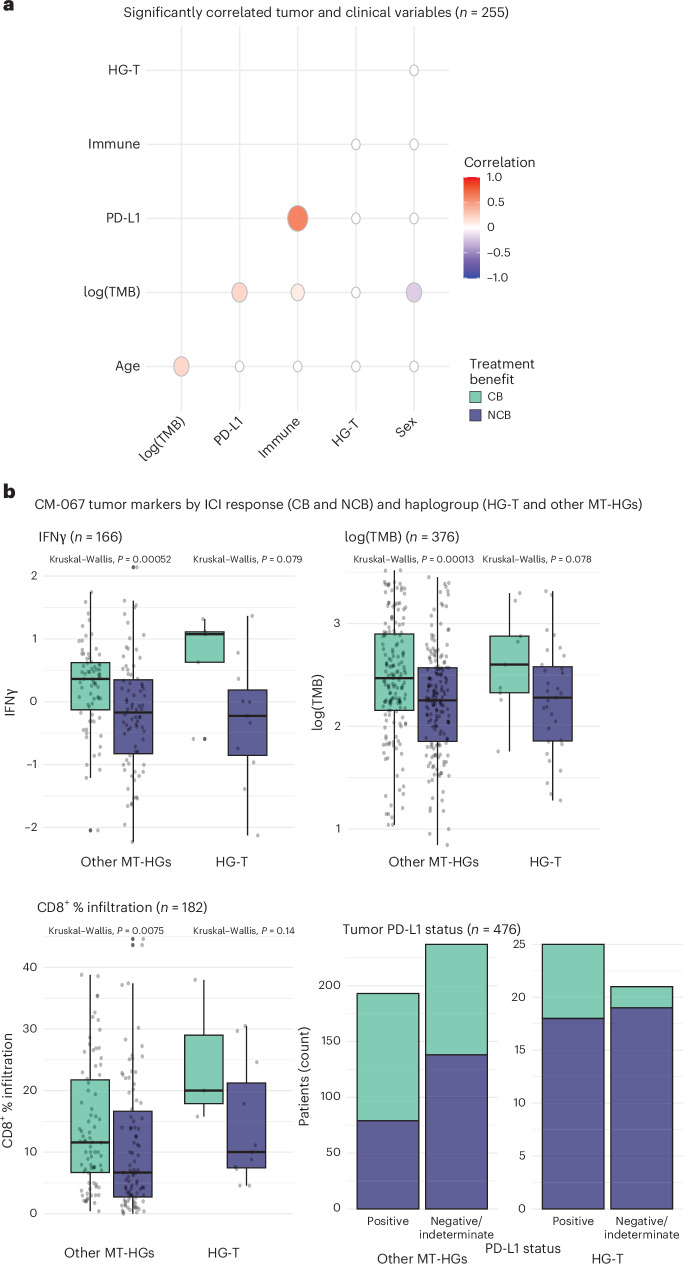
Tumor markers and haplogroups in CM-067. **a**, Correlation of tumor markers with clinical variables including log-transformed TMB, tumor PD-L1 status, composite ‘immune’ score (CD8^+^ T cell tumor infiltration > median, tumor IFNγ expression score > 0, or both), haplogroup (HG-T versus others) and sex (*n* = 255). **b**, Distribution of tumor markers stratified by haplogroup and ICI response for IFNγ score (*n* = 166), log-transformed TMB (*n* = 376), percentage tumor infiltration by CD8^+^ T cells (*n* = 182) and PD-L1 status (*n* = 476). Box plots depict lower and upper hinges (25th to 75th percentiles), median value (central line), whiskers extending from the hinges (largest and smallest values no more than 1.5 times the interquartile range) and outlying points outside this range (solid points beyond the whiskers). *P* values derived from Kruskal–Wallis tests. Source data

**Extended Data Fig. 3:**
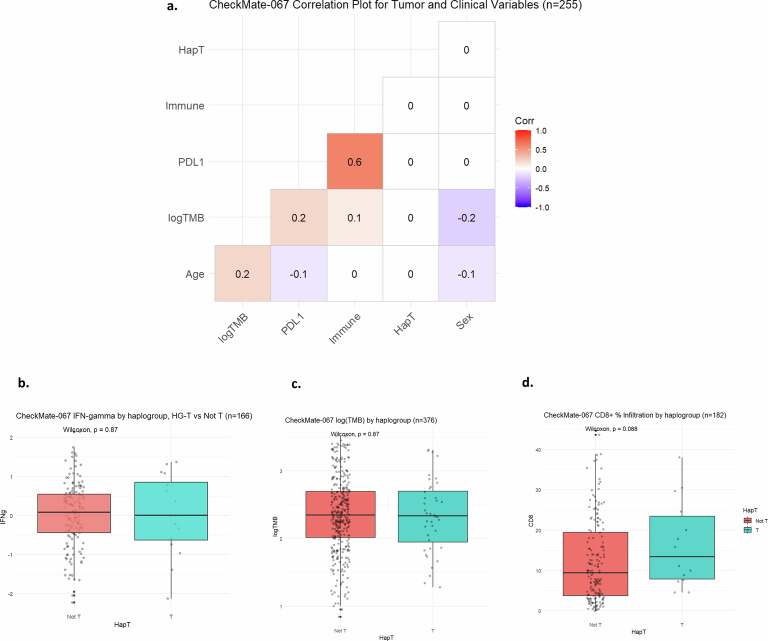
Association between HG-T and tumor markers in CM-067. **a**. Results of correlation analysis comparing HG-T to demographic and tumor characteristics (n=255): age, sex, log-transformed tumor mutation burden (TMB), tumor PD-L1 status, and tumor “immune” score (a composite variable for either positive IFN-γ gene expression, increased CD8^+^ tumor infiltration, or both). **b-d**. Distribution of tumor markers by haplogroup: **b**. IFN-γ score (n=166); **c**. log-transformed tumor mutation burden (TMB) (n=376); **d**. % Tumor Infiltration by CD8^+^ T cells (n=182). Boxplots depict lower and upper hinges (25th to 75th percentiles), median value (central line), whiskers extending from the hinges (largest and smallest values no more than 1.5 times the interquartile range), and outlying points outside this range (solid points beyond the whiskers). P-values derived from two-tailed Kruskal-Wallis tests.

We further tested whether HG-T may be a correlative for known tumor-based ICI predictors, or whether it represents an independent response biomarker. We evaluated the association between HG-T and CM-067 patients with tumor marker data available (*n* = 255 patients across the three treatment arms). This included PD-L1 status, CD8^+^ immune infiltration, TMB and expression of a ten-gene interferon-gamma (IFNγ) signature indicative of tumor inflammation (Supplementary Table 3). We calculated the correlation between HG-T, demographic variables (age and sex) and tumor variables (PD-L1 positivity; log_10_ TMB; and a composite score of either CD8^+^ T cell tumor infiltration > median, tumor IFNγ expression score > 0, or both; Fig. 3a). While PD-L1 status and the composite tumor immune infiltration score were highly positively correlated (*R* = 0.6), there were no other variables with a strong correlation, and HG-T was not correlated with any tumor or demographic factors (Fig. 3a and Extended Data Fig. 3a). When evaluating these tumor markers as continuous variables, we observed no difference in IFNγ expression (*P* = 0.87) or log_10_ TMB (*P* = 0.87); however, we have noted a slightly elevated CD8^+^ infiltration in HG-T compared to the other MT-HGs (*P* = 0.09; Extended Data Fig. 3b–d).

We next evaluated the association between tumor characteristics, MT-HGs and treatment response. For both HG-T and the other MT-HGs, patients with CB tended to have higher levels of each tested tumor marker (PD-L1, TMB, IFNγ/CD8^+^ immune infiltration; Fig. 3b). While each of these tumor markers is significantly associated with CB in NIVO, they do not predict response in COMBO. In contrast, HG-T alone significantly predicts poor outcomes in both NIVO and COMBO treatments.

### MT-HGs associated with baseline peripheral T cell phenotypes

Given the growing evidence that MT metabolism impacts T cell differentiation^16^, we assessed whether peripheral effector CD8^+^ T cells, as the targets of anti-PD-1 therapy, show distinct phenotypic features in patients with the NIVO-resistant haplogroup, HG-T, versus other haplogroups. To define a baseline immune profile associated with response and MT-HGs, we performed single-cell RNA sequencing (scRNA-seq) on pretreatment circulating CD8^+^ T cells from patients with HG-T and other MT-HGs.

We performed scRNA-seq analysis on CD8^+^ cells isolated from CM-067 NIVO-treated patients (*n* = 21), which included 8 patients with CB (*n* = 7 CR, *n* = 1 PR) and 13 with NCB (*n* = 13 PoD). To delineate the phenotypic differences associated with MT-HGs, we enriched the patient selection as follows: for NCB patients, we selected 7 HG-T (NCB-HG^T^) and an additional 6 NCB patients with other MT-HGs (NCB-HG°). For CB patients, we chose 1 with HG-T and 7 with other MT-HGs.

**Fig. 4:**
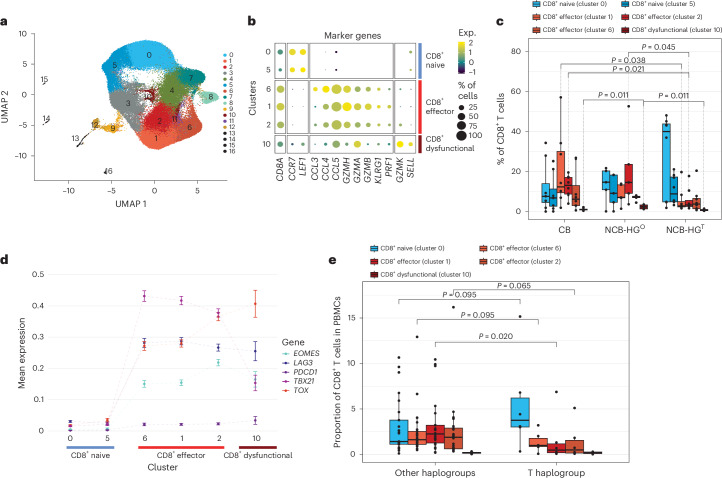
scRNA-seq analysis for pretreatment circulating CD8^+^ T cells from CM-067 NIVO patients (*n* = 21) and CM-915 patients (*n* = 31). **a**, UMAP dimensionality reduction plot (CM-067, *n* = 21 patients). **b**, Bubble plot showing per-cluster cell-type classification based on T cell marker gene expression (CM-067, *n* = 21 patients). **c**, Box plots showing single-cell CD8^+^ T cell-type proportions stratified by NIVO treatment response (CB, NCB) and haplogroup (HG^T^; other haplogroups, HG^O^; CM-067, *n* = 21 patients). *P* value from one-tailed Wilcoxon rank-sum test for the following comparisons: cluster 1, HG^T^ versus CB, *P* = 0.038; cluster 2, HG^T^ versus CB, *P* = 0.021, HG^T^ versus HG^O^, *P* = 0.045; cluster 10, HG^O^ versus HG^T^, *P* = 0.011, HG^O^ versus CB, *P* = 0.011. **d**, Scatterplot showing per-cluster mean expression of T cell exhaustion markers grouped by cell type. The connecting lines are used for the visualization of the changes in the gene expression of the selected markers per cluster. The clusters are ordered from least differentiated to exhausted phenotypes, based on the levels of differentiation markers as derived from **b**. **e**, Box plots showing single-cell CD8^+^ T cell-type proportions of baseline (postsurgical resection, pre-ICI) patient samples from CM-915 stratified by haplogroup (T haplogroup versus other haplogroups; CM-915, *n* = 31 patients). *P* value from one-tailed Wilcoxon rank-sum test, cluster 1, HG-T versus other MT-HGs, *P* = 0.020. Box plots depict lower and upper hinges (25th to 75th percentiles), median value (central line), whiskers extending from the hinges (largest and smallest values no more than 1.5 times the interquartile range) and outlying points outside this range (solid points beyond the whiskers). Source data

**Extended Data Fig. 4:**
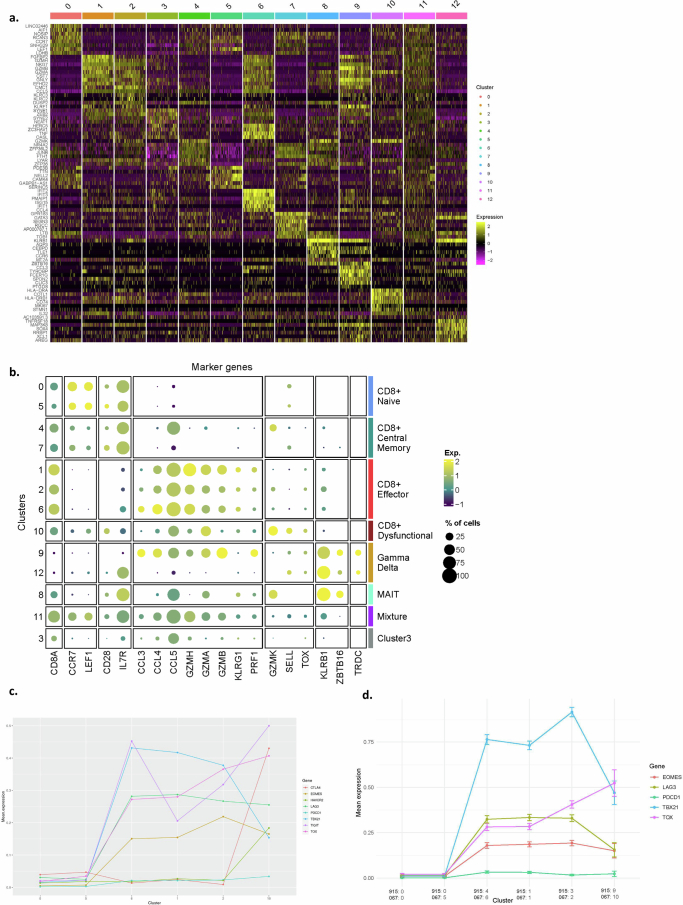
Cluster annotation and cell-type determination from CM-067 and CM-915 scRNA-seq. **a**. Top 8 significantly differentially expressed genes per cluster from single-cell (sc)RNA-seq of peripheral blood CD8^+^ T cells isolated from NIVO-treated CM-067 patients (n=21). **b**. Per-cluster cell-type classification based on differential expression of marker genes from scRNA-seq of peripheral blood CD8^+^ T cells isolated from NIVO-treated CM-067 patients (n=21). **c**. Per-cluster gene expression of T cell exhaustion markers from scRNA-seq of peripheral blood CD8^+^ T cells isolated from NIVO-treated CM-067 patients (n=21). **d**. Per-cluster gene expression of T cell exhaustion markers in CD8^+^ T cells from scRNA-seq of pre-treatment PBMCs from CM-915 patients (n=31). As this was an independent analysis, clusters are labeled by the cluster identified in CM-915 (top cluster number) and, based on their expression profiles, the corresponding cluster first identified in CM-067 (bottom cluster number).

Using established scRNA-seq pipelines^22–24^, we mapped the transcriptional profiles of the CD8^+^ T cells using the uniform manifold approximation and projection (UMAP) algorithm identifying 17 CD8^+^ T cell clusters (Fig. 4a). Based on differential gene expression profiles of T cell marker genes derived from established reference sets^24,25^, we annotated these 17 clusters to specific cell types (Extended Data Fig. 4a,b). We defined major phenotypic states used for downstream analysis, ordering them by T cell differentiation state (Fig. 4b): naive cells (clusters 0 and 5), effector cells (cluster 6, earliest stage of effector cell differentiation; cluster 1, early exhaustion; cluster 2, late exhaustion) and dysfunctional T cells (cluster 10), defined as those with the highest mean expression levels of exhaustion markers (Extended Data Fig. 4c).

When stratifying the CD8^+^ T cell phenotypes by NIVO response status and MT-HG, NCB-HG^T^ patients tended to have predominantly naive cells and significantly fewer early exhausted cells (cluster 1, *P* value = 0.02) and late exhausted cells (cluster 2, *P* value = 0.04) compared to CB patients. Compared to NCB-HG°, NCB-HG^T^ patients not only had fewer late exhaustion cells (cluster 2, *P* value = 0.05) but also significantly fewer dysfunctional cells (cluster 10, *P* value = 0.01; Fig. 4c). Of note, the one HG-T CB patient included in this analysis showed a phenotype similar to that of other CB patients, presenting with predominantly non-exhausted effector CD8^+^ T cells.

It has been shown that PD-1 inhibition can reinvigorate exhausted T cells and promote their effector function; however, only a subset of exhausted cells, characterized by high T-bet (*TBX21*^hi^) and low EOMES (*EOMES*^lo^), is capable of reinvigoration^26,27^. Conversely, cells that continue along this differentiation trajectory become terminally exhausted and cannot be reinvigorated^25,28,29^; these cells are characterized by low T-bet (*TBX21*^lo^), high EOMES (*EOMES*^hi^) and high TOX (*TOX*^hi^). Based on this model, we further refined exhausted phenotypes identified in our data and defined *TBX21*^hi^ exhausted cells, the primary target of reinvigoration by NIVO, and those with high *EOMES* (*EOMES*^hi^) and/or *TOX* (*TOX*^hi^). Cluster 1, with *TBX21*^hi^ and *EOMES*^lo^ expression (Fig. 4d), likely represents the early exhaustion phenotype that can be reinvigorated and is predominantly present in CB patients. NCB-HG° patients show elevated levels of both cluster 2 (*EOMES*^hi^, *TOX*^hi^ and *TBX21*^lo^) and cluster 10 (*EOMES*^lo^, *TBX21*^lo^ and *TOX*^hi^; Fig. 4d), both likely the terminally exhausted cells (cluster 2) or dysfunctional T cells (cluster 10) with no capacity for anti-PD-1 reinvigoration. In contrast, NCB-HG^T^ patients show a significantly lower proportion of all three states of exhausted cells, exhibiting a distinct baseline peripheral cell phenotype characterized by an overall lower fraction of differentiated CD8^+^ T cells in patients with these haplogroups.

To further validate the HG-T baseline peripheral phenotype, we generated scRNA-seq data from PMBCs of an additional *n* = 31 patients available from a separate clinical trial (CheckMate-915 (CM-915)). These included *n* = 6 patients with HG-T and *n* = 25 patients of other non-T MT-HGs. Based on the gene expression profiles from this independent analysis, we were able to match the corresponding clusters of CD8^+^ T cells identified from the CM-915 scRNA-seq with those defined in CM-067 (Extended Data Fig. 4d). This comparison further confirmed the overall reduction in terminally exhausted T cells in patients belonging to HG-T (Fig. 4e) compared to other non-T MT-HGs. The association was statistically significant in the same direction as in CM-067 patients (*P* = 0.0203), further confirming that this is a peripheral cell phenotype specific to HG-T.

### HG-T CD8^+^ cells express ROS detoxification genes

The results from scRNA-seq analysis in CM-067 demonstrated the three patterns of baseline CD8^+^ T cell phenotypes stratified by both treatment response (CB/NCB) and MT-HG status (HG-T versus other MT-HGs). The most distinct subgroups were HG-T NCB patients (with very few differentiated effector cells) and NCB patients with other MT-HGs (with a higher fraction of terminally exhausted ‘dysfunctional’ effector cells). Existing evidence suggests that the mitochondrially driven ROS production drives effector T cell differentiation^16^, and there are prior observations indicating that ROS tolerance may differ between HG-T and other MT-HGs^17^. We therefore sought to identify whether transcriptional differences of ROS or other related metabolic pathways between HG-T and non-T HGs may expand further functional understanding of how MT-HGs associate with the T cell phenotypes observed in the scRNA-seq analysis.

**Fig. 5:**
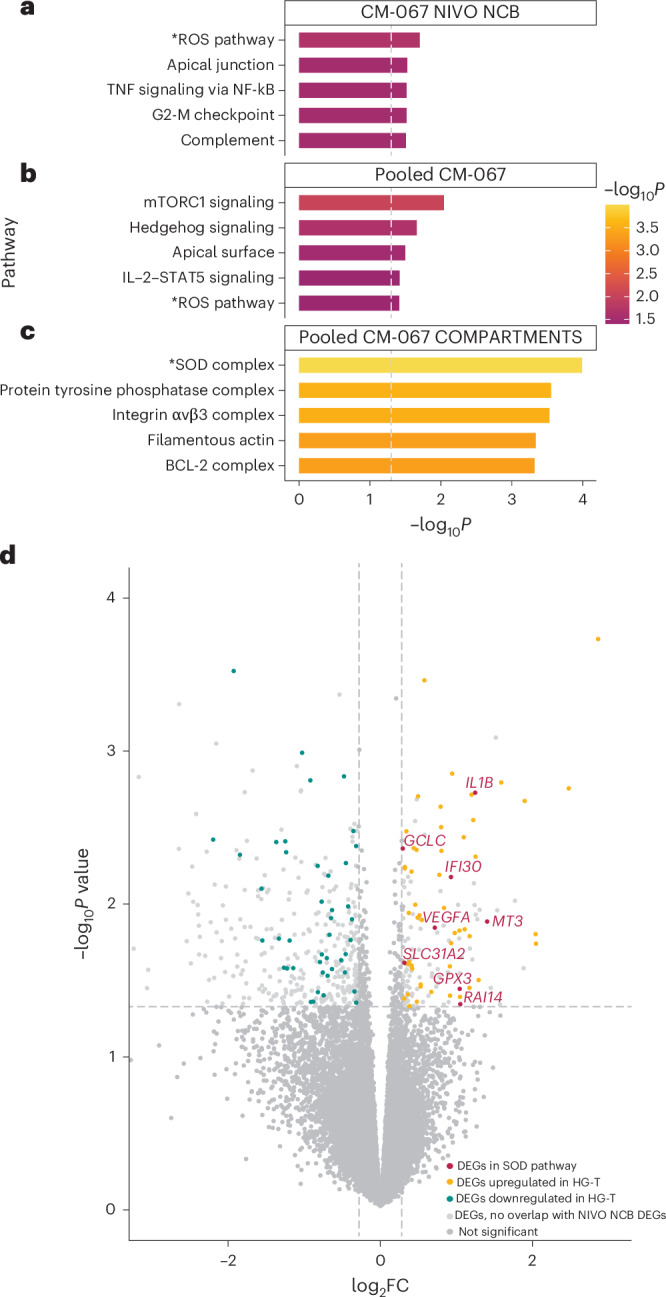
Baseline differences in gene expression by haplogroup comparing bulk RNA-seq from peripheral blood CD8^+^ T cells in CM-067. **a**, Top five significantly enriched pathways from GO analysis on top DEGs from CM-067 NIVO NCB cohort (*n* = 62 patients) comparing HG-T (*n* = 9) versus other MT-HGs (*n* = 53). −log_10_-transformed *P* values derived from two-tailed Fisher’s exact test. **b**,**c**, Top five significantly enriched pathways from GO analysis (Fig. 5b) and COMPARTMENTS database (Fig. 5c) on top DEGs from CM-067 NIVO and COMBO cohorts (*n* = 212 patients) comparing HG-T (*n* = 17) versus other MT-HGs (*n* = 195). −log_10_-transformed *P* values derived from two-tailed Fisher’s exact test. **d**, Volcano plot from differential expression analysis from NIVO and COMBO CM-067 analysis (*n* = 212) showing upregulation of DEGs in the SOD pathway in HG-T. Genes with negative log_2_ fold change (log_2_FC) are downregulated in HG-T; genes with positive log_2_FC are upregulated in HG-T. Red points are the significant DEGs in both NIVO NCB and NIVO and COMBO analyses that are present in the SOD pathway. Yellow and blue points are DEGs that are significantly upregulated and downregulated, respectively, in HG-T in both NIVO NCB and NIVO and COMBO analyses. Light gray points are significant DEGs in the NIVO and COMBO analysis that did not overlap with DEGs in the NIVO NCB comparison. Dark gray points are genes that were not significantly differentially expressed and did not overlap between the NIVO NCB comparison and NIVO and COMBO comparison. −log_10_-transformed *P* values derived from negative binomial Wald test in DESeq2. Source data

**Extended Data Fig. 5:**
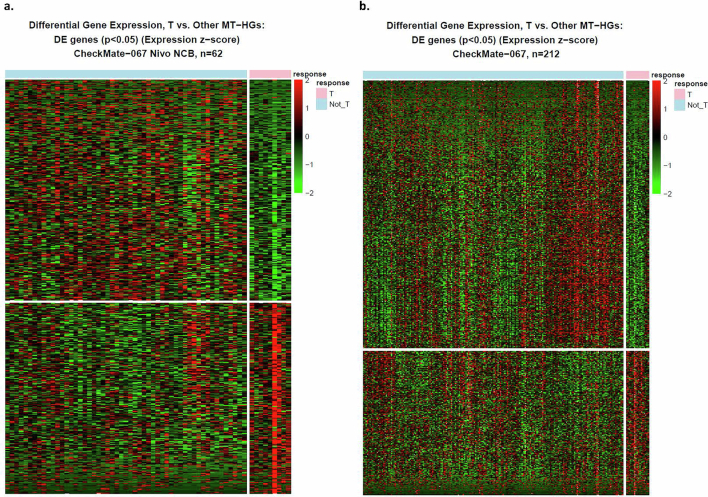
Differentially expressed genes from CM-067 bulk RNA-seq analysis of circulating CD8^+^ T cells comparing HG-T and other MT-HGs. Heatmaps showing upregulated (red) and downregulated (green) genes for HG-T (pink) or other MT-HGs (blue) among **a**. NIVO NCB patients (n=62) and **b**. cohort of NIVO and COMBO CM-067 patients with bulk RNA-sequencing regardless of outcome (n=212).

We performed bulk RNA-seq analysis on CD8^+^ T cells isolated from NIVO-treated NCB patients from CM-067 (*n* = 62; *n* = 9 with HG-T and *n* = 53 with other MT-HGs). Differential gene expression analysis demonstrated baseline transcriptional differences in the immune cells of patients with different haplogroups: 240 differentially expressed genes (DEGs) were upregulated in HG-T, and 277 were upregulated in the other MT-HGs (Extended Data Fig. 5a). Consistent with our hypothesis, Gene Ontology (GO) analysis on the top 260 DEGs revealed the ROS pathway as the top significantly enriched pathway (*P* = 0.02; Fig. 5a), with two genes (glutamate-cysteine ligase catalytic subunit, *GCLC*; and glutathione peroxidase 3, *GPX3*) associated with ROS detoxification upregulated in HG-T.

To test the baseline differences between HG-T and other non-T HGs, regardless of treatment outcome, we performed another DEG analysis on a larger cohort of CM-067 patients treated with either NIVO or COMBO and comprising both CB and NCB outcomes (total *n* = 212 CM-067 patients; *n* = 17 HG-T, *n* = 195 other MT-HGs; Extended Data Fig. 5b). Using the overlap between the top DEGs from the NIVO NCB analysis and the DEGs in this comparison, we identified 127 of 206 genes still differentially expressed, all with the same directionality as in the original analysis. When performing GO on this set of 127 genes, the ROS pathway was still among the top five significantly enriched pathways (*P* = 0.04; Fig. 5a), with *GCLC* and *GPX3* again upregulated in HG-T. We further explored the pathways enriched among these DEGs using the COMPARTMENTS database, which uses protein subcellular localization evidence to explore the potential cellular functions of proteins. Here, the most significantly enriched pathway was the superoxide dismutase (SOD) complex pathway (*P* = 1.02 × 10^−4^; Fig. 5a), involving genes encoding proteins related to key antioxidant cellular functions^30^. The SOD pathway contains both *GCLC* and *GPX3* (also found in the ROS pathway) but also includes additional antioxidant-related genes found to be upregulated in HG-T, including *SLC31A2*, *IL1B*, *IFI30*, *MT3*, *RAI14* and *VEGFA* (Fig. 5b).

### Association between MT-HG and post-ICI survival

**Fig. 6:**
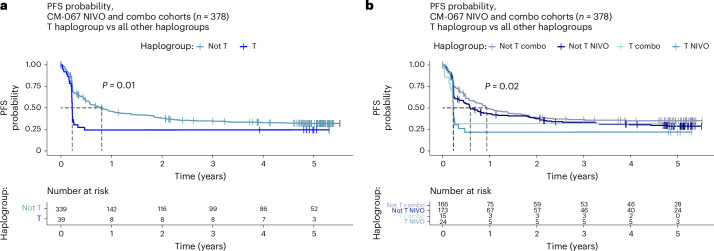
PFS probability by haplogroup in CM-067. **a**, PFS (in years) comparing HG-T (*n* = 39) and other MT-HGs (*n* = 339) in CM-067 NIVO and COMBO cohorts (*n* = 378). *P* value from two-tailed Kaplan–Meier (log-rank) test. **b**, PFS (in years) stratified by treatment, comparing HG-T treated by NIVO (*n* = 24) and COMBO (*n* = 15) and other MT-HGs treated by NIVO (*n* = 173) and COMBO (*n* = 166). *P* value from two-tailed Kaplan–Meier (log-rank) test. Source data

**Extended Data Fig. 6:**
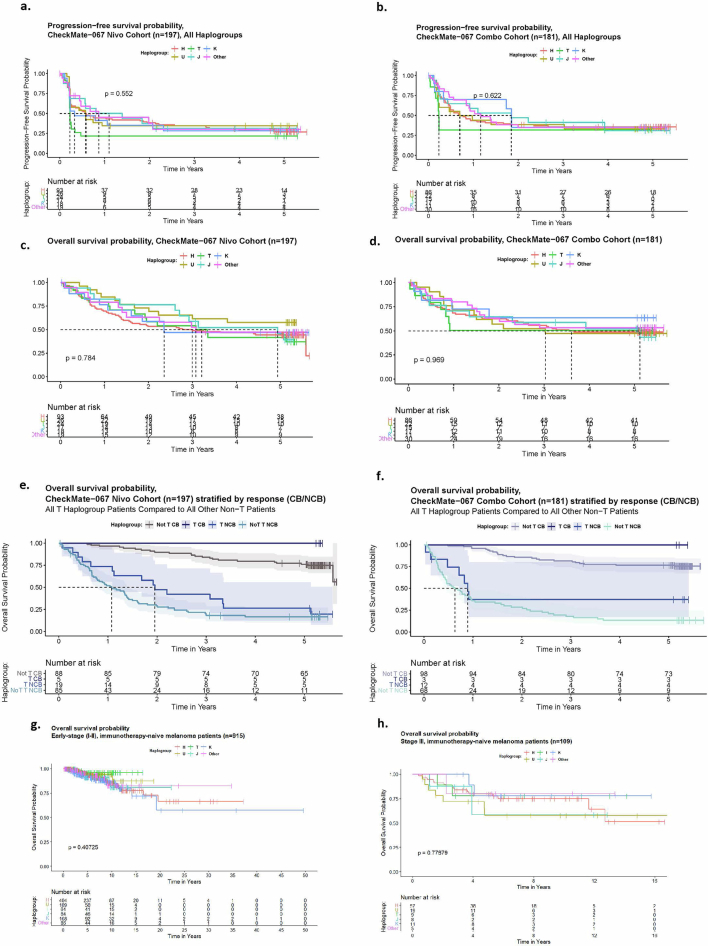
Progression-free and overall survival stratified by MT-HG in CM-067 NIVO cohort (n=197) and COMBO cohort (n=181), and IMCG prognostic cohort. **a-b**. Progression-free survival (PFS) stratified by MT-HG in CM-067 **a**. NIVO and **b**. COMBO cohorts. **c-d**. Overall survival (OS) stratified by MT-HG in CM-067 **c**. NIVO and **d**. COMBO cohorts. **e-f**. OS stratified by MT-HG (HG-T vs Other MT-HGs) and response in CM-067 **e**. NIVO and **f**. COMBO cohorts. Light bands represent 95% confidence intervals. **g-h**. OS by MT-HG in IMCG prognostic cohort for **g**. early-stage (Stage I-II) disease (n=915) and **h**. late-stage (Stage III) disease (n=109). All p-values from two-tailed Kaplan-Meier (log-rank) test.

As survival is the most definitive indicator of ICI therapy success, we also tested whether MT-HGs are associated with progression-free survival (PFS) and overall survival (OS) after ICI (Extended Data Fig. 6a–d and Supplementary Tables 4 and 5a,b) using well-harmonized survival data from CM-067. We found significantly decreased PFS among the HG-T NIVO-treated patients (age, sex, stage-adjusted PFS hazard ratio = 1.83 (95% CI: 1.09–3.05), *P* value = 0.021) and a similar trend for COMBO-treated patients (adjusted PFS hazard ratio = 1.78 (95% CI: 0.89–3.57), *P* value = 0.11; Fig. 6a,b and Supplementary Table 4). Indeed, 78% of all patients belonging to HG-T progressed within the first 4 months of treatment (75% for NIVO-treated HG-T and 80% for COMBO-treated HG-T), and their OS was also significantly poorer than patients who experienced CB (NIVO adjusted *P* value < 0.001; COMBO adjusted *P* value < 0.001), and comparably poor to all other patients with NCB (Extended Data Fig. 6e,f and Supplementary Table 5c,d).

### MT-HGs predict ICI outcome independent of disease prognosis

Given the observed associations of HG-T with NIVO and COMBO resistance, validated in multiple patient populations in our study, we sought to evaluate whether MT-HG status is predictive of ICI outcomes or a reflection of overall disease prognosis. We assessed the association of MT-HGs and OS in a prognostic cohort of 1,024 ICI-naive patients with melanoma (not treated by ICI) enrolled at New York University Langone Health^31^, with extensive follow-up (Extended Data Fig. 6g,h). In this analysis, we found no difference in survival in early-stage (I and II) patients (*n* = 915, *P* value for OS by haplogroup = 0.41; Extended Data Fig. 6g and Supplementary Table 5e) or later-stage (III) patients in both univariable and multivariable analyses (*n* = 109, *P* value for OS by haplogroup = 0.78; Extended Data Fig. 6h and Supplementary Table 5f).

## Discussion

Growing evidence points to the important role of mitochondrially driven metabolism in T cell activation, differentiation and host antitumor effector T cell capacity, leading to its emergence as an attractive new research area in immuno-oncology. It is plausible that interindividual variability of mitochondria-mediated antitumor immunity is impacted by genetic variability in mtDNA, which may be an important factor contributing to ICI efficacy. In this study, we have tested and validated the association of inherited MT-HGs with ICI efficacy in melanoma, providing a link between MT inheritance and ICI-mediated antitumor immune response.

Analyzing the MT genome of 1,225 ICI-treated patients with MM, we found that ICI efficacy varies by MT-HGs. We discovered that HG-T is significantly associated with resistance to NIVO (pooled OR = 3.46), which we validated in both a clinical trial (CM-067) as well as in an independent SOC ICI cohort from the IO-GEM. This is, to our knowledge, the first inherited genetic marker predicting patients before treatment who will not benefit from single-agent NIVO, a treatment option with otherwise high efficacy and low toxicity. In alignment with these observations, NIVO-resistant patients belonging to HG-T show poor PFS and OS, when compared to other MT-HGs treated by single-agent NIVO.

Our study also showed that patients belonging to HG-T experience comparably poor outcomes with anti-PD-1/anti-CTLA-4 combination therapy (COMBO; 34% objective response rate, OR = 3.64), which is also a frontline ICI therapy in MM. With no statistical difference observed in anti-CTLA-4 efficacy between HG-T and other MT-HGs, these findings collectively indicate that HG-T is specifically associated with resistance to NIVO-based treatments (single-agent or in combination with anti-CTLA-4), and this also correlates with significantly poorer survival (Fig. 6 and Extended Data Fig. 6). The integration of the observed associations with established tumor-based predictors, including tumor PD-L1 expression, TMB and IFNγ score, is of particular clinical importance. We found no significant difference between HG-T and the other MG-HGs in the distribution of any of the studied tumor markers. We noted, as expected, more favorable tumor characteristics (for example, PD-L1 positivity, increased TMB and immune score) in patients with CB, which also included HG-T responders. This implies that, for the small fraction of patients belonging to HG-T who will eventually respond to NIVO-based treatments, the presence of positive predictive tumor markers may be indicative of CB. However, even with positive tumor markers, the patients with HG-T are less likely to respond compared to the other MT-HGs with favorable tumor indicators. While NCB patients in general had less favorable tumor predictors, we did not find significant differences in tumor characteristics between HG-T and other haplogroups among patients with NCB. Collectively, these results suggest that HG-T, as a predictor of nonresponse to NIVO-based treatments, is independent from the known tumor-based indicators and thus may represent a biomarker with stand-alone clinical value for predictive testing.

**Extended Data Fig. 7:**
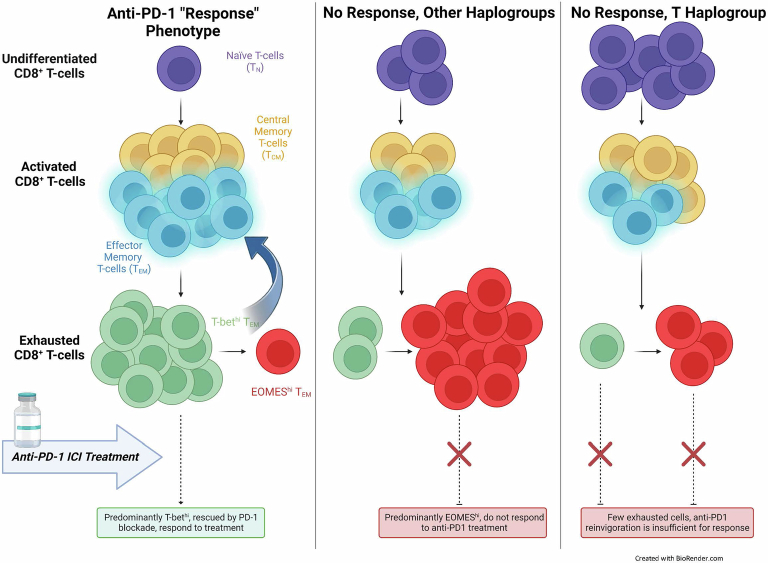
Summary of circulating pre-treatment CD8^+^ T cell phenotypes defined using single-cell (sc)RNA-seq on patients from BMS-067 NIVO (n=21). 1. Clinical Benefit (CB) “response” phenotype: predominantly T cells able to be reinvigorated by anti-PD-1. 2. No Clinical Benefit in haplogroups other than HG-T (NCB-O): predominantly dysfunctional or terminally exhausted effector cells unable to be reinvigorated by anti-PD-1. 3. No Clinical Benefit in HG-T (NCB-T): few cytotoxic or exhausted effector cells, anti-PD-1 reinvigoration is insufficient. Created with BioRender.com.

The study outlines a possible new link between MT inheritance, host immune cell repertoire and ICI efficacy. It has been proposed that PD-1 inhibition returns the cytotoxic capacities of certain phenotypes of exhausted CD8^+^ T cells, possibly improving antitumor immunity. In contrast, dysfunctional cells (terminally exhausted) can no longer be reinvigorated by PD-1 inhibition^26–28^. Our scRNA-seq data show that the peripheral baseline distribution of these exhausted cell phenotypes varies by MT-HG and treatment efficacy in NIVO-treated patients. We have identified three distinct peripheral CD8^+^ effector T cell exhaustion phenotypes by NIVO response and MT-HG status (summarized in Extended Data Fig. 7). We observed that patients with CB exhibit higher baseline levels of cell phenotypes capable of regaining cytotoxic activity by NIVO reinvigoration. In contrast, patients with NCB of non-T MT-HGs show a lesser fraction of these cells, but more terminally exhausted and dysfunctional phenotypes, no longer able to be reinvigorated by NIVO. Finally, NCB patients with HG-T show significantly lower overall baseline levels of all differentiated CD8^+^ T cell states, either terminally exhausted/dysfunctional or those that can be reinvigorated by PD-1 inhibition. This NIVO resistance-associated CD8^+^ T cell phenotype possibly points to an altered differentiation cascade, which, in patients belonging to HG-T, retains CD8^+^ T cells at earlier differentiation stages. These observations suggest the preexisting host immune cell repertoire associated with HG-T status, which we validated in an independent cohort of patients with melanoma from the CM-915 trial, identifies patients belonging to HG-T as having a reduced proportion of baseline peripheral CD8^+^ effectors T cells. This indicates that patients belonging to HG-T present with a specific baseline host immune phenotype, as a property of host immunity, potentially altering the capacity of NIVO-induced antitumor response.

Given these findings, we hypothesized that MT-HG status may be associated with the processes that drive peripheral CD8^+^ T cells into effector states and that this capacity affects response to ICI. While the biological mechanisms explaining these correlations are elusive, they are consistent with recent studies showing that T cell differentiation to effector states depends on differential tolerance to ROS^16^, which varies by MT-HGs. The published data have reported that HG-T can better tolerate ROS compared to other MT-HGs. Upon PD-1 blockade, ROS also stimulate the AMPK–mTOR pathway, leading to an increase in T-bet production and a decrease in EOMES^32^. Based on this evidence, in individuals with greater tolerance to ROS, such as those with HG-T, more ROS may be needed to drive the cells into the activated state. This may explain why we observed very few effector cells in the CD8^+^ repertoire of patients belonging to HG-T not responding to NIVO.

Interestingly, the results of our bulk RNA-seq on pretreatment circulating CD8^+^ T cells, comparing patients belonging to HG-T versus those of non-T MT-HGs, provide a potential functional explanation for the differences in ROS tolerance by different MT-HGs. Our data revealed ROS and SOD as the most significantly enriched pathways among the DEGs, including *GCLC*, *GPX3*, *IL1B*, *IFI30*, *VEGFA* and *MT3*, *s*pecifically pointing to the upregulation of ROS detoxification in patients belonging to HG-T. This provides a possible mechanistic insight suggesting that it is in fact the higher level of ROS detoxification in patients belonging to HG-T that reduces the ROS-mediated T cell differentiation, likely explaining the distinct peripheral CD8^+^ T cell repertoires in patients with NCB belonging to HG-T. In particular, the genes upregulated in patients belonging to HG-T center around the synthesis and activity of glutathione^33^, an established critical driver of the primary cellular defense against ROS^34^. The baseline transcription of some of these genes, for example, *IL1B*, has previously shown differences among MT-HGs in cybrid models^35^, further supporting the role of mtDNA variation modulating nuclear expression of ROS clearance pathways. Collectively, these results support a putative role of glutathione-mediated ROS detoxification in patients belonging to HG-T. As demonstrated by the overall lack of effector cells in patients belonging to HG-T, we hypothesize that this increased ROS scavenging may contribute to a decrease in ROS-mediated T cell differentiation in these patients, which will need to be evaluated in further metabolic models on different MT backgrounds.

Our results linking MT-HGs and response to ICI support the involvement of mitochondria-associated ROS in T cell differentiation. This highlights the need for more focused biological investigations connecting MT genetics with ROS metabolism and T cell antitumor immunity, eventually with therapeutic implications, which we are currently pursuing. While the findings in this study implicate the effect of HG-T on peripheral T cell differentiation, it is also possible that the effect of MT-HGs will be apparent in the T cell compartment of the tumor microenvironment, which may provide a further biological link between mitochondria and tumor T cell contexture before ICI treatment. Using the available tumor infiltration data from CM-067, we found some indication of increased pretreatment (baseline) CD8^+^ T cell tumor infiltration in HG-T compared to the other MT-HGs; however, these observations were of borderline significance (*P* = 0.09). Given that there was no association between HG-T and either the IFNγ expression score^36^ or the composite ‘inflamed tumor’ score (assessing increased CD8^+^ infiltration, IFNγ expression or both), these findings suggest that HG-T status per se does not predict an inflamed tumor phenotype.

Taken together, HG-T, as a biomarker of ICI resistance independent of other tumor characteristics, may highlight opportunities for other ICI treatment combinations that patients belonging to HG-T may benefit from. This includes the recent development of new combination ICIs that incorporate blockade of other immune checkpoints (for example, LAG-3, TIGIT and TIM-3) or alternative immunotherapies such as recently approved adoptive cell therapies in melanoma^37^. While not tested in this study, with the availability of sufficiently powered cohorts allowing for MT-HG testing with adequate reproducibility, opportunities for further stratification benefits for patients belonging to HG-T to other treatments may become possible.

Strengths of our study include the large sample size (*n* = 1,225 ICI-treated patients), with a discovery and validation dataset generated from well-controlled clinical trial samples (CM-067) and a SOC treatment cohort from a multinational study (IO-GEM), ensuring the rigor and reproducibility of the findings. Combining these cohorts generated the largest genomic analysis to date of ICI-treated patients with MM to explore the role of inherited MT genetics in ICI efficacy. Because HG-T represents a germline biomarker, we can determine MT-HGs at any timepoint, allowing us to analyze any available tissue in biobanks, as well as publicly available data, mitigating a potential bias in patient selection when specimens are collected retrospectively. Further, unlike previous studies that combine patients into one ‘ICI-treated’ population, stratifying by treatment allowed us to analyze outcomes separately by ICI therapy as well as by haplogroup. The addition of scRNA-seq data is another strength, extending the observational findings to identify a relevant peripheral host cell phenotype connecting MT-HGs with a putative biological model of HG-T impacting ROS resistance and ICI efficacy.

While, to our knowledge, this study is one of the largest to date exploring germline genetic predictors of MM ICI efficacy, promoting this biomarker to further clinical utility will require additional prospective assessment in a larger patient cohort, in both SOC and potentially in a dedicated clinical trial for biomarker validation. Such independent evaluation can also address with more granularity the association with long-term survival, which will require a substantially larger sample size with well-annotated long-term follow-up clinical information. Among other study limitations is that we were only able to assess patients treated with NIVO and/or IPI; while these are currently accepted SOC treatments for MM, we plan to test this biomarker in alternative treatments and combinations when resources from these clinical trials, with sufficient statistical power, are available. Another limitation is that the scRNA-seq analyses did not assess dynamic on-treatment changes, which is an objective of current research endeavors. While our data provide some initial evidence of the potential predictive capacity of other rare MT-HGs (for example, we saw a trend for potential NIVO resistance associated with HG-K and NIVO sensitivity associated with HG-J), future studies in larger patient populations are required to confirm these associations. Finally, this study assessed only ICI-treated patients with MM, who are predominantly of white European ancestry. Nevertheless, given that HG-T represents a host immune inherited biomarker detected in pretreatment peripheral immune cells, it is conceivable that its clinical relevance may be broadly applicable beyond melanoma. As such, our efforts to assess the predictive capabilities of MT-HGs in other ICI-treated cancers and other non-European populations are currently underway.

Our study provides, to our knowledge, the first evidence that MT-HG status is associated with ICI outcomes. MT-HG genetic assessment may represent an independent, clinically viable and minimally invasive baseline biomarker that can identify patients who are less likely to respond to current NIVO-based treatment regimens. From a clinical standpoint, there are a substantial number of patients with favorable tumor predictors, either PD-L1-high and/or TMB-high, and yet do not respond to SOC ICI treatment, requiring further clinical stratification. This biomarker, which based on our findings appears to be independent of these tumor indicators, may further predict which of these patients with otherwise favorable tumor markers will be resistant to prescribed therapies. Possibly, adding another layer of information, such as scRNA-seq from peripheral immune cells, may further improve the stratifying capacity of MT-HGs, in particular for the rare subset of patients belonging to HG-T who still respond to NIVO-based ICI therapies. Pending further prospective clinical validation, this study underscores the importance of rational approaches for adding independent surrogates with stand-alone clinical predictive value in more personalized treatment recommendations. Further, we highlight the connection between MT genetics and the peripheral blood immune cell repertoire in the context of ICI resistance. This link reveals new avenues for biological exploration of the interplay between genetic control of mitochondria and ICI-mediated antitumor immunity, collectively paving the path toward the biological understanding of MT relevance in immuno-oncology.

## Methods

### Study population

**Extended Data Fig. 8:**
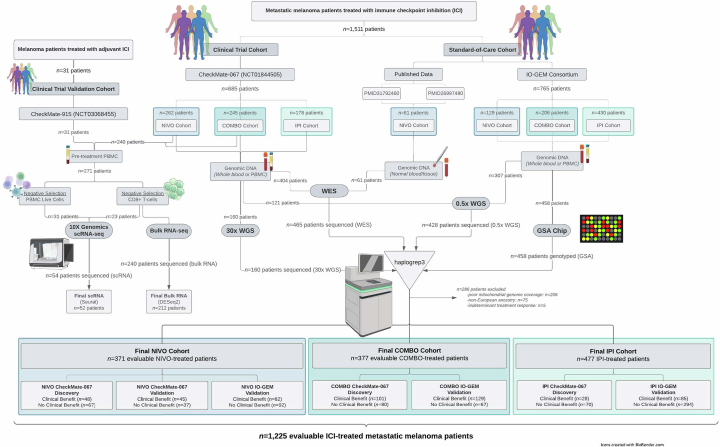
Study population flow diagram. Flow diagram illustrating the patient population, sample collection, multi-omic material extraction, sequencing/genotyping, and haplogroup determination for the analytic cohorts. Created with BioRender.com.

In this study, we used patient samples from the CM-067 clinical trial (ClinicalTrials.gov registration: NCT01844505), a three-arm phase III study that enrolled participants with therapy-naive advanced melanoma who were treated between 2013 and 2015 with single-agent IPI, NIVO or the combination (COMBO or IPI-NIVO; Extended Data Fig. 8)^1,2^. The CM-067 protocol was approved by the institutional review boards (IRBs) of all participating study sites. In the present CM-067 analysis, we sequenced pretreatment specimens of peripheral blood mononuclear cells (PBMCs) or purified peripheral CD8^+^ T cells from *n* = 155 NIVO and *n* = 126 COMBO patients and utilized previously generated data from whole-blood-based whole-exome sequencing (WES) of patients treated by NIVO, COMBO and IPI (*n* = 454). In total, *n* = 526 CM-067 patient samples were used in this analysis. The best overall response to treatment in CM-067 was defined as CR, PR, SD or PoD, based on Response Evaluation Criteria in Solid Tumors (version 1.1)^1^. CB was defined as patients with CR, PR or dSD (stable disease without progression for at least 6 months). Patients with NCB were defined as PoD or SD who progressed within 6 months (pSD). We additionally performed sensitivity analyses comparing only patients with objective response, defined as those with a best overall response of CR or PR, to those with PoD. Patients were followed for 60 months to assess OS and PFS^1^.

For validation of the findings from CM-067, we used the IO-GEM Consortium, a SOC cohort of ICI-treated patients with melanoma from centers in the United States, Europe and Australia. Samples for this analysis were collected at the following institutions and the sample and data collection protocols were approved by each institution’s IRB and patient informed consent was obtained before collections: New York University Langone Health (NYULH IRB), Memorial Sloan Kettering Cancer Center (MSK)(Memorial Sloan Kettering IRB), University of California Los Angeles’s Jonsson Comprehensive Cancer Center (UCLA-JCCC; University of California Los Angeles IRB), Massachusetts General Hospital (MGH), Dana Farber Cancer Institute (DFCI; both under Dana Farber Cancer Institute IRB), University of Chicago Comprehensive Cancer Center (UCCCC; The University of Chicago IRB), University of Colorado Cancer Center (CUCC; Colorado Multiple IRB), Roswell Park Cancer Institute (RPCI; Roswell Park Cancer Institute IRB and Scientific Review Board) and the National Tumor Institute Fondazione G. Pascale in Naples, Italy (INT-IRCCS; The Ethics Committee of National Cancer Institute IRCCS Fondazione G. Pascale). All patients had MM treated with NIVO, COMBO or IPI (Extended Data Fig. 8). Each patient provided blood samples (whole blood or PBMCs) for DNA extraction. We also obtained data from publicly available sequencing of ICI-treated patients with MM^19,20^. In total, the SOC validation cohort consisted of *n* = 675 patients. Response status was determined by the centers and was similarly dichotomized into CB (CR, PR and dSD) and NCB (pSD and PoD). Patients with SD for whom we could not determine the duration of their SD were removed from the analysis (*n* = 5).

For prognostic analyses of survival stratified by haplogroups, we used a cohort of immunotherapy-naive patients with melanoma who were prospectively enrolled in the Interdisciplinary Melanoma Cooperative Group (IMCG) at NYULH from 2002 to 2018 (*n* = 1,024 participants)^31^. Clinicopathological, follow-up and biospecimen collection protocols for the IMCG have been previously described^31,38,39^. For confirmatory analysis of the expected MT-HG population distribution, we obtained MT-HGs from *n* = 420 participants from the European populations in the 1000 Genomes Project (1kGP)^40^. Genomic data for this analysis were collected from 1kGP phase 1 (*n* = 244 participants) and phase 3 (*n* = 176 participants).

To validate the immune cell phenotypes identified in scRNA-seq analysis of peripheral CD8^+^ T cells from CM-067, we used pretreatment PBMC samples from the CM-915 clinical trial (ClinicalTrials.gov registration: NCT03068455), a two-arm phase III study of adjuvant treatment with either NIVO or combined IPI-NIVO (COMBO), enrolling patients within 12 weeks of complete surgical resection of stage III B/C/D or IV melanoma^41^. The CM-915 protocol was approved by the IRB at all participating study sites.

### Sequencing and genotyping

#### 30× WGS sample preparation and data processing

We isolated genomic DNA from pretreatment PBMCs from CM-067 using the Qiagen Blood and Tissue Kit. Samples were processed using the Illumina TruSeq DNA PCR-Free kit (for samples with DNA content >500 ng) or the New England BioLabs NEBNext Ultra II DNA Library Prep Kit for Illumina (for samples with <500 ng DNA) per manufacturers’ instructions (Illumina; New England BioLabs). Samples were sequenced to a target of 30× genomic coverage on an Illumina HiSeq X or NovaSeq 6000 per manufacturer’s instructions, using 2 × 150-bp paired-end reads.

**Extended Data Fig. 9:**
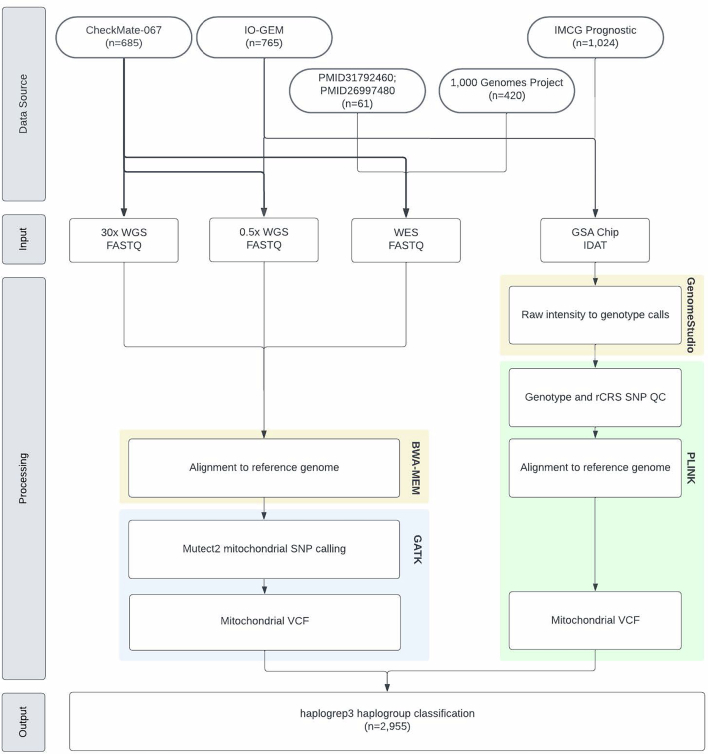
Genomics data analysis workflow. Workflow for computationally determining mitochondrial haplogroups.

Genomic data analysis pipelines were performed as summarized in Extended Data Fig. 9 using Genome Analysis Toolkit (GATK) best practices^42^. Raw FASTQ files were aligned to the Genome Reference Consortium Human Build 38 reference genome (GRCh38) using bwa ‘mem’ (version 0.7.17). The resulting BAM files were merged using Sambamba ‘merge’ (version 0.6.8), sorted with Samtools ‘sort’ (version 1.9) and duplicates marked using Sambamba ‘markdup’ and indexed using samtools ‘index’. We then performed two rounds of base quality score recalibration using ‘BaseRecalibrator’ (version 4.1.2.0) from the GATK^42^. To identify variants in the MT genome, variant call format files (VCFs) were generated by GATK’s Mutect2 (version 4.2.1.0) using ‘mitochondria-mode’ which is designed for sensitive variant detection at high sequencing depth. Finally, MT-HGs were classified by passing the MT VCF files to Haplogrep 3 (version 3.2.1)^43^. To define the MT-HGs, we used the MT phylogenetic tree from Phylotree (version 17.1)^44^ aligned to the revised Cambridge Reference Sequence (rCRS) by specifying ‘--tree’ phylotree-rcrs@17.1 in the Haplogrep ‘classify’ statement.

We performed quality control (QC) to ensure sufficient sequencing depth and sample identity. We used the samtools ‘index’ function on the aligned BAM files to index and split them each by chromosome. The read depth at each position and the number of reads were measured by Samtools ‘depth’ and Samtools ‘view’ separately. The per-base coverage was then calculated using the formula: Coverage = (number of reads × read length) / total genome size. The mean MT genome coverage for this analysis was 2,259× (mean autosomal coverage 26.0×).

#### Ultra-low-coverage (0.5×) WGS sample preparation and data processing

As we have shown previously^45^, ultra-low-coverage WGS (lcWGS; approximately 0.5× average genomic coverage) is sufficiently effective for analysis of common genetic variation (>5% of the population) when compared to both 30× WGS and chip-based genotyping methods like the Global Screening Array (GSA). An additional 121 CM-067 samples and 307 IO-GEM samples were sequenced using lcWGS. Genomic DNA was isolated as described above (see ‘30× WGS sample preparation and data processing’). Before sequencing, samples were identity-tracked using a panel of 25 previously curated variants^13^ on the Sequenom MassArray System (Agena Bioscience), per the manufacturer’s instructions. lcWGS sequencing libraries were prepared using either the NEBNext Ultra II DNA Library Prep Kit for Illumina or the Illumina DNA Prep kit, including up to eight cycles of PCR. Libraries were quantified using real-time PCR and sequenced on a NovaSeq 6000 per the manufacturer’s instructions using 2 × 150-bp paired-end reads. We observed high concordance (>99%) between the identity panel genotyping and the sequenced lcWGS samples, minimizing the likelihood of possible sample mismatches.

lcWGS FASTQ files were processed into BAM files according to the GATK workflow as described above (see ‘30× WGS sample preparation and data processing’) and reads were aligned to the GRCh37 reference genome. Genotype likelihoods were estimated with bcftools ‘mpileup’ (version 1.9) using the 1kGP phase 3 reference panel, and imputation was performed using GLIMPSE (version 1.0.0). Following imputation, MT-HGs were determined as above (see ‘30× WGS sample preparation and data processing’). Mean lcWGS MT genome coverage was 121× (mean autosomal coverage 0.69×).

#### Infinium GSA sample preparation and data processing

MT-HGs were determined for a subset of the IO-GEM SOC cohort and the immunotherapy-naive IMCG cohort using the Infinium Global Screening Array Multi-Disease (GSA-MD) platform (Illumina). Genomic DNA was isolated from whole blood or PBMCs using the Qiagen DNeasy 96 Blood and Tissue Kit. Before genotyping, samples were identity-tracked as described above and, similarly, we observed high concordance (>99%) between the identity panel genotyping and the GSA-MD, minimizing the likelihood of sample mismatches.

Using Illumina’s GenomeStudio (version 2.0), raw intensity data (.idat) files from the GSA-MD were converted to genotype calls with a GenCall score threshold of 0.15 and exported them to PLINK^46^ format for downstream analysis using the PLINK Input Report Plug-in (version 2.1.4). Genotype QC was performed in PLINK (version 1.9/2.0) by first excluding SNPs not present in ≥20% of the samples (assumed to be poorly genotyped SNPs) using the ‘*--*geno’ command and excluding any patients missing ≥20% of the remaining SNPs using the ‘*--*mind’ command (assumed to be patients with poor overall genotype quality).

As MT-HGs are defined by the specific nucleotide at each position, SNPs in the Haplogrep 3 input VCF file must be reported according to the rCRS reference. GSA probes can target sequences on either the forward or the reverse DNA strand; SNPs called from probes on the forward strand are reported according to the rCRS, while those on the reverse strand are reported as the complementary base. We therefore identified all the MT SNPs with GSA probes on the reverse strand and translated them to the complementary base using the ‘--flip’ command in PLINK. Using the ‘--exclude’ PLINK command, we additionally dropped several MT SNPs that were retained after the ‘--geno’ filter but which are not well covered on the GSA. We then generated BED files aligned to the GRCh37 reference genome using the ‘--ref-from-fa’ PLINK command. Finally, we converted the BED file to a VCF file using the ‘--recode vcf’ PLINK command and passed this to Haplogrep 3, including the ‘--chip’ argument for sequencing array data.

#### WES

For the additional CM-067 patients not included in lcWGS or WGS sequencing (*n* = 454), we obtained off-target MT sequences^47^ from pretreatment matched-normal whole-blood WES^48^. To obtain the MT-HG information, we processed the raw WES FASTQ files using the same pipeline as the WGS samples described above (see ‘30× WGS sample preparation and data processing’). Additional publicly available WES data were obtained from the SOC setting from *n* = 61 ICI-treated patients with MM from published data^19,20^. MT-HGs were assessed using the off-target WES reads as described. To determine MT-HGs from the 1kGP, we obtained existing haplogroup data from phase 1 and derived haplogroup data from phase 3 WES as outlined above.

#### MT-HG concordance

To ensure concordance between the sequencing modalities used in this analysis, we compared Haplogrep 3 results from each platform for a subset of patients. We observed high MT-HG concordance across all platforms, with 100% concordance between ×30 and ×0.5 WGS, 97% concordance between WGS and GSA, and 100% concordance between WES and lcWGS.

#### Tumor marker analyses

For a subset of the patients in the CM-067 clinical trial, tumor-based biomarkers were available^48^. Immunohistochemistry was used to determine tumor PD-L1 status (≥5% versus <5% or indeterminate) and the percentage of tumor CD8^+^ T cell infiltration (percentage of CD8^+^ immune cells of total cells). TMB was calculated as the sum of somatic missense mutations and was either log_10_ transformed (when assessed as a continuous variable) or dichotomized into high (>median) or low (≤median) TMB. A ten-gene IFNγ signature was calculated as the median of *z*-score-transformed normalized gene expression for *HLA-DRA*, *CXCL9*, *GZMA*, *PRF1*, *CCR5*, *IFNG*, *CXCL10*, *IDO1*, *STAT1* and *CXCL11*, with a range of −1 (low expression) to 1 (high expression)^36^. Because not all variables were available for all patients, we created a composite score for ‘increased tumor immunogenicity’ wherein patients were deemed to have increased tumor immune infiltration if they had a CD8^+^ score ≥ median percentage, IFNγ score > 0, or both. Correlations between tumor markers and clinical variables were computed using the R package ‘ggcorrplot’.

#### scRNA-seq and data analysis

##### CM-067

CD8^+^ T cells were negatively selected from pretreatment PBMCs from 23 NIVO-treated patients using the EasySep Human CD8^+^ Enrichment Kit (STEMCELL Technologies) per the manufacturer’s instructions. Single-cell suspensions for each patient were individually tagged with unique hashtag antibodies (TotalSeq-B Anti-Human antibodies, BioLegend). Four patient samples were pooled as one sample in suspension (*n* = ~12,500 cells per hashtag, *n* = ~50,000 total cells per pool) for a total of six pooled samples. Single cells were partitioned into Gel Beads-in-Emulsion (GEMs) using the Chromium Next GEM Chip G and tagged with a common 10x barcode and a unique molecular identifier (10x Genomics, Chromium Next GEM Single Cell 3′ GEM Library & Gel Bead Kit). Reverse transcription, amplification and library preparation of 3′ gene expression libraries were performed per established protocols (10x Genomics). Libraries were sequenced using the NovaSeq 6000 platform at a minimum of 20,000 read pairs per cell at the Perlmutter Cancer Center Genome Technology Center Shared Resource (GTC).

scRNA-seq QC and downstream analysis of CD8^+^ T cells were performed with the Seurat v3 R toolkit^22–24^. Demultiplexing of samples based on hashtag oligonucleotides and annotation of doublets removed from the analysis was performed using the ‘HTODemux’ function with default parameters. Cells with gene counts >200 and <12,500 were kept for downstream analysis, while genes expressed in <3 cells were filtered out. In total, 92,531 CD8^+^ T cells from 21 patients successfully passed QC and were used for the present analysis. Batch correction was then performed in Seurat using the integration method to mitigate artifactual differences between sequencing pools. Normalization of the data was performed using ‘NormalizeData’ with the ‘LogNormalize’ method and the most variable genes were found using the ‘FindVariableGene’ function with ‘nfeatures’ set to 2,000. Principal component analysis (PCA) was run on 50 dimensions and after a visual inspection of the elbow plot, the first 15 principal components were chosen to construct a shared nearest-neighbor graph with the ‘FindNeighbors’ function. ‘FindClusters’ was applied to identify clusters with the resolution set to 0.5. The UMAP algorithm was used to visualize the clustering in 2D space.

##### CM-915

Additional scRNA-seq was performed using pretreatment PBMC samples from 31 patients on the CM-915 clinical trial. Negative selection was performed to enrich samples for live cells using the EasySep Dead Cell Removal (Annexin V) Kit (STEMCELL Technologies) per the manufacturer’s instructions. To increase library complexity, single-cell suspensions for each patient sample were divided in half, and a unique hashtag antibody was assigned to each half (TotalSeq-C Anti-Human antibodies, BioLegend). To detect rarer immune cell subsets within the total PBMC population, two patient samples were pooled in one suspension, resulting in pools of two patients (*n* = ~30,000 cells per patient, *n* = ~60,000 total cells per pool). Sample sequencing and data analysis were then performed as described above for the CM-067 samples.

#### Bulk RNA-seq and data analysis

Pretreatment PBMCs were used as a source of CD8^+^ T cell populations from 240 patients. The CD8^+^ cells were negatively selected from the overall PBMC population using an EasySep Human CD8^+^ Enrichment Kit (STEMCELL Technologies) per the manufacturer’s instructions, and 100,000–200,000 viable CD8^+^ cells were used for RNA extraction using the AllPrep DNA/RNA kit for simultaneous purification of genomic DNA and total RNA from human cells (Qiagen). The purified RNA specimens were quantified using Qubit RNA High Sensitivity Assay (Thermo-Fisher Scientific), with Bioanalyzer High Sensitivity Analysis (Agilent Technologies) of the sample’s RNA integrity number serving to assess sample quality. Purified samples were stored at −80 °C until sequencing.

The SMARTer Stranded Total RNA-Seq Kit v2 Pico Input Mammalian (Takara Bio USA) was used to prepare rRNA-depleted RNA-seq libraries. Each library was quantified by qPCR and libraries were pooled for sequencing at Azenta Life Sciences using 150-bp paired-end sequencing to a depth of ~30 million reads per sample. FastQC was used for QC of the FASTQ files, and sequencing adaptors were removed from the raw RNA-seq reads using fastp^49^. STAR aligner (v2.7)^50^ was used to map and align the reads to the human reference genome (median paired reads per sample of 28,470,039 across all 240 samples, minimum 12 million reads per sample) and gene expression and transcript quantification was obtained using RSEM^51^.

Using the R package DESeq2, we assessed baseline differential gene expression levels between patients of different MT-HGs. After normalization in DESeq2, the PCA plots were used to identify sequencing outliers (>5 s.d. from PCA mean) before analysis (*n* = 17 sequencing outliers dropped from the analysis). We then evaluated differences in gene expression between patients with HG-T compared to other MT-HGs, first among only CM-067 NIVO NCB, then all CM-067 patients for whom we performed bulk RNA-seq; genes with a *P* value < 0.05 were considered differentially expressed. To find the genes most likely involved in well-defined biological pathways, only nuclear-encoded protein-coding DEGs were used in GO analyses.

### Statistical analysis

Differences in treatment outcomes by haplogroup were assessed using non-parametric chi-squared tests, likelihood-ratio tests and logistic regression models. Likelihood-ratio test *P* values are reported, as they are robust to variations in sample size. We compared patients with CB versus those with NCB and analyzed NIVO-treated, IPI-treated and COMBO-treated cohorts separately. In the IO-GEM cohort, we also conducted a sensitivity analysis assessing the association between MT-HG and patients with any NIVO treatment (NIVO as first-line or second-line ICI therapy) and those with first-line NIVO ICI treatment only (ICI treatment-naive). Using the R package ‘pwr’ (version 1.3-0), we performed a power analysis to evaluate the magnitude of the effect detectable in our analytic cohorts. Given the sample size of both the CM-067 and IO-GEM patient populations, we achieved 98% power in the NIVO cohort and 94% power in the COMBO cohort to detect a calculated effect size (*h*) for two proportions of *h* = 0.6 at an *α* = 0.1.

MT-HG phylogenetic trees were constructed using the R package ‘ape’. Forest plots for logistic regression ORs were plotted using the R package ‘gt’. Survival analysis was conducted using the R package ‘survival’, with Kaplan–Meier curves calculated using the ‘survfit’ function and Cox proportional hazards models generated using ‘coxph’. Pooled statistical models were adjusted for clinical parameters including age, self-reported sex and disease stage when available. For a subset of patients for whom age was unknown (*n* = 99), we performed five iterations of Multiple Imputation by Chained Equations using the ‘mice’ package in R. For each patient with missing age, the average of their imputed ages was used for covariate analyses. Cox proportional hazard assumptions were confirmed using the Schoenfeld residuals test. A two-tailed *P* value of <0.05 was considered statistically significant. All analyses were conducted in R version 4.2.2.

### Reporting summary

Further information on research design is available in the Nature Portfolio Reporting Summary linked to this article.

## Online content

Any methods, additional references, Nature Portfolio reporting summaries, source data, extended data, supplementary information, acknowledgements, peer review information; details of author contributions and competing interests; and statements of data and code availability are available at 10.1038/s41591-025-03699-3.

## Supplementary information

Reporting Summary

Supplementary Tables 1–5.

## Source data

Source Data Figs. 1–6 Statistical source data for Figs. 1, 2, 3, 4c,e, 5a–d and 6.

## Data Availability

The data generated on industry-sponsored clinical trial specimens and SOC patient specimens (IO-GEM) used in this publication are not publicly available due to restrictions protecting patient identity, unless otherwise stated below. Currently, there does not exist consent that would allow public sharing of any genomic or other omic data generated from these specimens. However, upon reasonable inquiry, the data requests may be directed to the corresponding author. The data requests will be reviewed by the study sponsor before fulfillment. Data from different centers will be shared differently according to variable local regulatory requirements. Those de-identified data that are not readily shared will be made available upon reasonable request and provided in accordance with corresponding regulatory requirements. Data from Fondazione ‘G. Pascale’ of Naples are available in a public, open-access repository at Zenodo via 10.5281/zenodo.10807767 (ref. ^52^). The publicly available data used in this study are available at dbGaP (accession number phs000452.v3.p1) or the Sequence Read Archive (accession numbers SRP067938 and SRP090294). Source data are provided with this paper.
