# Supplementary material for: Inherited mitochondrial genetics as a predictor of immune checkpoint inhibition efficacy in melanoma
Source: Nat Med. 2025 Jun 5;31(7):2385–96. doi: 10.1038/s41591-025-03699-3 (PMC12283385; doi:10.1038/s41591-025-03699-3)
Supplement: Supplementary file 1 — Reporting Summary [file 41591_2025_3699_MOESM1_ESM.pdf]

nature portfolio

Corresponding author(s): Tomas Kirchhoff

Last updated by author(s): Mar 17, 2025

Reporting Summary

Nature Portfolio wishes to improve the reproducibility of the work that we publish. This form provides structure for consistency and transparency in reporting. For further information on Nature Portfolio policies, see our Editorial Policies and the Editorial Policy Checklist.

Statistics

For all statistical analyses, confirm that the following items are present in the figure legend, table legend, main text, or Methods section.

n/a	Confirmed	
☐	☒	The exact sample size (*n*) for each experimental group/condition, given as a discrete number and unit of measurement
☐	☒	A statement on whether measurements were taken from distinct samples or whether the same sample was measured repeatedly
☐	☒	The statistical test(s) used AND whether they are one- or two-sided<br>*Only common tests should be described solely by name; describe more complex techniques in the Methods section.*
☐	☒	A description of all covariates tested
☐	☒	A description of any assumptions or corrections, such as tests of normality and adjustment for multiple comparisons
☐	☒	A full description of the statistical parameters including central tendency (e.g. means) or other basic estimates (e.g. regression coefficient) AND variation (e.g. standard deviation) or associated estimates of uncertainty (e.g. confidence intervals)
☐	☒	For null hypothesis testing, the test statistic (e.g. *F*, *t*, *r*) with confidence intervals, effect sizes, degrees of freedom and *P* value noted<br>*Give P values as exact values whenever suitable.*
☒	☐	For Bayesian analysis, information on the choice of priors and Markov chain Monte Carlo settings
☒	☐	For hierarchical and complex designs, identification of the appropriate level for tests and full reporting of outcomes
☐	☒	Estimates of effect sizes (e.g. Cohen's *d*, Pearson's *r*), indicating how they were calculated

Our web collection on statistics for biologists contains articles on many of the points above.

Software and code

Policy information about availability of computer code

Data collection	No software for data collection has been used
Data analysis	Genomic analyses were conducted using bwa (version 0.7.17), sambamba (version 0.6.8), samtools (version 1.9), BaseRecalibrator (version 4.1.2.0), Mutect2 (version 4.2.1.0), haplogrep3 (version 3.2.1), bcftools (version 1.9), GLIMPSE (version 1.0.0), GenomeStudio (version 2.0), and PLINK (version 1.9 and 2.0). Data analyses were conducted in R (version 4.2.2) using the following packages: Seurat (version 3), DESeq (version 1.42.1), ggcorrplot (version 0.1.4.999), pwr (version 1.3-0), ape (version 5.8-1), gt (version 0.11.1), survival (version 3.6-4), and mice (version 3.17.0).

For manuscripts utilizing custom algorithms or software that are central to the research but not yet described in published literature, software must be made available to editors and reviewers. We strongly encourage code deposition in a community repository (e.g. GitHub). See the Nature Portfolio guidelines for submitting code & software for further information.

Data

Policy information about availability of data

All manuscripts must include a data availability statement. This statement should provide the following information, where applicable:

- Accession codes, unique identifiers, or web links for publicly available datasets
- A description of any restrictions on data availability
- For clinical datasets or third party data, please ensure that the statement adheres to our policy

The data generated on industry-sponsored clinical trial specimens used in this publication are not publicly available due to restrictions protecting patient identity. Currently, there does not exist consent that would allow public sharing of any genomic or other omic data generated from these specimens. However, upon reasonable inquiry, the data requests may be directed to the corresponding author. The data requests will be reviewed by the study sponsor prior to fulfillment. Data from different centers will be shared differently according to different local regulatory requirements. Those deidentified data that are not readily shared will be made available upon reasonable request and provided in accordance with corresponding regulatory requirements. Data from Fondazione “G. Pascale” of Naples are available in a public, open-access repository at the following external link https://zenodo.org/record/10.5281/zenodo.10807767
The publicly available data used in this study is available at dbGaP (accession number phs000452.v3.p1) or the Sequence Read Archive (accession numbers SRA: SRP067938 and SRA: SRP090294)

Research involving human participants, their data, or biological material

Policy information about studies with human participants or human data. See also policy information about sex, gender (identity/presentation), and sexual orientation and race, ethnicity and racism.

Reporting on sex and gender	Demographic tables reporting the distribution of self-reported sex are reported for each analysis/dataset
Reporting on race, ethnicity, or other socially relevant groupings	Demographic tables reporting the distribution of ethnicity are reported for each analysis/dataset
Population characteristics	Demographic tables describing the population characteristics are reported for each analysis/dataset, including age, sex, and mitochondrial haplogroup. Additional CM-067 characteristics were also available and are reported, including disease stage at study entry, tumor BRAF and PD-L1 status, tumor mutation burden, IFN-gamma score, and tumor % CD8 infiltration.
Recruitment	Ascertainment details, collection of patient specimens are described per each respective cohort
Ethics oversight	Ethics oversight as related to the study participant is provided per each respective cohort/center. The sample and data collection protocols were approved by each institution’s Institutional Review Board (IRB) and patient informed consent was obtained prior to collections: New York University Langone Health (NYULH)(New York University Langone Health IRB), Memorial Sloan Kettering Cancer Center (MSK)(Memorial Sloan Kettering IRB), University of California Los Angeles’s Jonsson Comprehensive Cancer Center (University of California Los Angeles IRB), Massachusetts General Hospital (MGH), Dana Farber Cancer Institute (DFCI)(both under Dana Farber Harvard Cancer Center IRB), University of Chicago Comprehensive Cancer Center (UCCCC)(The University of Chicago IRB), University of Colorado Cancer Center (CUCC)(Colorado Multiple Institutional Review Board; COMIRB), Roswell Park Cancer Institute (RPCI)(Roswell Park Cancer Institute IRB and Scientific Review Board), and the National Tumor Institute Fondazione G. Pascale in Naples, Italy (INT-IRCCS) (The Ethics Committee of National Cancer Institute IRCCS Fondazione “G. Pascale”, Napoli, Italy).

Note that full information on the approval of the study protocol must also be provided in the manuscript.

Field-specific reporting

Please select the one below that is the best fit for your research. If you are not sure, read the appropriate sections before making your selection.

☒ Life sciences ☐ Behavioural & social sciences ☐ Ecological, evolutionary & environmental sciences

For a reference copy of the document with all sections, see nature.com/documents/nr-reporting-summary-flat.pdf

Life sciences study design

All studies must disclose on these points even when the disclosure is negative.

Sample size	N=l,225. We used R package “pwr” (v.1.3-0) to perform a power analysis to evaluate the magnitude of the effect detectable in our analytic cohorts. Given the sample size of both the CheckMate-067 and IO-GEM patient populations, we achieved sufficient 98% power in the NIVO cohort and 94% power in the COMBO cohort to detect a calculated effect size (h) for two proportions of h=0.6 at an α=0.1.
Data exclusions	Patients of non-European ancestries, with poor sequencing quality or insufficient clinical data were excluded (n=307)
Replication	The study has used discovery and validation sample cohorts. In NIVO analyses: One discovery and one replication cohort for CM-067 (n=115, n=82, respectively), a fully independent replication standard-of-care (IO-GEM) cohort (n=174). All replication attempts were successful. For COMBO and IPI: one discovery cohort in CM-067 (n=181, n=98, respectively) and one fully independent IO-GEM cohort (n=196, n=379,

respectively). For COMBO, all replication was successful for CM-067 and the comparable effect size was observed for IO-GEM replication. For scRNA analysis, one discovery cohort from CM-067 (n=21) and one independent validation cohort from CM-915 (n=31). For scRNA-seq all replication attempts was successful.

Randomization: CM-067 and CM-915 were randomized as per described protocol, IO-GEM samples were collected regardless of treatment outcome, as described in the manuscript.

Blinding: The study was not blinded. This is an observational study to identify new genetic (inherited) markers associated with ICI outcomes, which would not be possible in the blinded fashion. The patients were assigned to treatment based on clinical decisions (IO-GEM) or randomly (CM-067).

Reporting for specific materials, systems and methods

We require information from authors about some types of materials, experimental systems and methods used in many studies. Here, indicate whether each material, system or method listed is relevant to your study. If you are not sure if a list item applies to your research, read the appropriate section before selecting a response.

Materials & experimental systems

n/a	Involved in the study	
☐	☒	Antibodies
☒	☐	Eukaryotic cell lines
☒	☐	Palaeontology and archaeology
☒	☐	Animals and other organisms
☒	☐	Clinical data
☒	☐	Dual use research of concern
☒	☐	Plants

Methods

n/a	Involved in the study	
☒	☐	ChIP-seq
☒	☐	Flow cytometry
☒	☐	MRI-based neuroimaging

Antibodies

Antibodies used: TotalSeq-B and -C anti-Human antibodies (BioLegend) were used for hash-tagging scRNA-seq samples for patient tracing as part of a standard protocol

Validation: N/A

Plants

Seed stocks: *Report on the source of all seed stocks or other plant material used. If applicable, state the seed stock centre and catalogue number. If plant specimens were collected from the field, describe the collection location, date and sampling procedures.*

Novel plant genotypes: *Describe the methods by which all novel plant genotypes were produced. This includes those generated by transgenic approaches, gene editing, chemical/radiation-based mutagenesis and hybridization. For transgenic lines, describe the transformation method, the number of independent lines analyzed and the generation upon which experiments were performed. For gene-edited lines, describe the editor used, the endogenous sequence targeted for editing, the targeting guide RNA sequence (if applicable) and how the editor was applied.*

Authentication: *Describe any authentication procedures for each seed stock used or novel genotype generated. Describe any experiments used to assess the effect of a mutation and, where applicable, how potential secondary effects (e.g. second site T-DNA insertions, mosiacism, off-target gene editing) were examined.*
